# Albumin levels and risk of early cardiovascular complications after endovascular thrombectomy for acute ischaemic stroke

**DOI:** 10.1038/s41598-026-50280-0

**Published:** 2026-04-24

**Authors:** Michele Rossi, Muath Alobaida, Enrico Tartaglia, Mert Kaskal, Amir Askarinejad, Andrea Galeazzo Rigutini, Steven Ho Man Lam, Tommaso Bucci, Garry McDowell, Gregory Y. H. Lip

**Affiliations:** 1 Liverpool Centre for Cardiovascular Science at University of Liverpool, Liverpool John Moores University and Liverpool Heart and Chest Hospital, Liverpool, UK; 2 Department of Life, Health and Environmental Sciences, University of L’Aquila, L’Aquila, Italy; 3 Internal Medicine and Nephrology Division, ASL1 Avezzano-Sulmona-L’Aquila, San Salvatore Hospital, L’Aquila, Italy; 4 Department of Basic Science, Prince Sultan Bin Abdulaziz College for Emergency Medical Services, King Saud University, Riyadh, Saudi Arabia; 5 Division, Department of Biomedical, Metabolic and Neural Sciences, University of Modena and Reggio Emilia, Policlinico di Modena, Modena, Italy; 6 Internal, Vascular and Emergency Medicine – Stroke Unit, University of Perugia, Perugia, Italy; 7 Department of Clinical Internal, Anaesthesiologic and Cardiovascular Sciences, Sapienza University of Rome, Rome, Italy; 8 School of Pharmacy and Biomolecular Sciences, Liverpool John Moores University, Liverpool, UK; 9 Danish Center for Health Services, Department of Clinical Medicine, Aalborg University, Aalborg, Denmark; 10 Department of Cardiology, Lipidology and Internal Medicine with Intensive Coronary Care Unit, Medical University of Bialystok, Bialystok, Poland

**Keywords:** Endovascular thrombectomy, Albumin, Ischemic stroke, Stroke-heart syndrome, Cardiovascular outcomes, Biomarkers, Cardiology, Diseases, Medical research, Neurology

## Abstract

We evaluate the clinical course of patients with hypoalbuminemia who underwent endovascular thrombectomy (EVT) within 24 h of acute ischaemic stroke (AIS). A retrospective cohort study using data from the global federated research network TriNetX. Adults ≥ 18 years treated with EVT for AIS between 2018 and 2024 with serum albumin measured within 24 h post-EVT were included. Patients were grouped by serum albumin levels: reduced (≤ 3.4 g/dL) vs. normal (≥ 3.5 g/dL). The primary outcome was a 30-day composite of all-cause death, acute heart failure, atrial fibrillation, ventricular arrhythmias, acute myocardial infarction, Takotsubo syndrome. Secondary outcomes included individual components and intracranial haemorrhage. Propensity score matching (1:1) balanced covariates, followed by Cox regression to estimate hazard ratios (HRs) and 95% confidence interval (CI)s. Among 8,698 patients with AIS treated with EVT, 6,233 had normal serum albumin (mean age 67.0 ± 14.5 years, 44.0% female), while 2,465 had reduced albumin (69.7 ± 14.3 years, 51.5% female). After matching, 2,374 patients with reduced albumin had higher risk of the composite outcome (HR 1.28, 95%CI 1.17–1.39), all-cause mortality (HR 1.72, 95%CI 1.50–1.96), and acute myocardial infarction (HR 1.74, 95%CI 1.28–2.35), compared to those with normal albumin. These associations remained consistent across subgroups stratified by age, sex, stroke severity, causes of hypoalbuminemia, degrees of hypoalbuminemia reduction. Hypoalbuminemia in patients with AIS treated with EVT is associated with an increased risk of early cardiovascular events. Serum albumin may be a prognostic biomarker in this population.

**Supplementary Information:**

The online version contains supplementary material available at 10.1038/s41598-026-50280-0.

## Introduction

Cardiac complications are the second leading cause of post-stroke mortality, affecting 10%–20% of patients within the first few days after a stroke^1,2^. Cardiac events occurring within 30 days of acute stroke are clinically termed stroke–heart syndrome (SHS), driven by several pathophysiological mechanisms, with inflammation playing a central role^3^. Following stroke, excessive release of catecholamines, cortisol and proinflammatory cytokines can impair cardiomyocytes function, primarily through microvascular injury^3,4^. This can result in contraction band necrosis, reduced myocardial contractility, and increased susceptibility to arrhythmias. SHS may present as myocardial infarction, heart failure, arrhythmias, or Takotsubo syndrome secondary. Importantly, stroke survivors who develop new cardiovascular complications have over a 50% risk of recurrent stroke within five years^5^.

Given the inflammatory and oxidative nature of SHS, biomarkers with anti-inflammatory and antioxidant properties may provide valuable prognostic information. Albumin, the most abundant circulating protein, is vital for molecule transport, maintenance of colloidal osmotic pressure, and also possesses anti-inflammatory^6,7^ and antioxidant properties^8^. Previous observational studies in post-stroke cohorts have shown that reduced serum albumin levels (≤ 3.4 g/dL) are associated with increased risks of mortality and cardiovascular events^9–11^. These findings suggest that albumin could serve as a marker of systemic vulnerability or diminished physiological reserve. However, limited evidence is available on the prognostic significance of albumin in patients undergoing endovascular thrombectomy (EVT), a subgroup characterized by large vessel occlusion and a high baseline risk of adverse outcomes. Even after successful EVT, these patients may experience an increased inflammatory response resembling ischemia/reperfusion injury, potentially amplifying cardiovascular risk^12^.

The study aimed to evaluate the associations between serum albumin levels and the 30-day risk of cardiovascular events or death in a large cohort of ischaemic stroke patients treated with EVT, using data from a global federated research network.

## Methods

This retrospective cohort study used data from TriNetX, a global federated health research network “Global Collaborative network” aggregating de-identified electronic medical records from 151 participating health care organisations, primarily in the United States, and others in the United Kingdom, Germany, Italy, Israel, and Singapore. This database includes patient demographics, diagnoses, procedures, medications, and laboratory results^13^. Diagnoses and treatments were identified using ICD-10, RxNorm, VA Drug Classification, and CPT codes.

### Study cohort

Data were queried on 13th March 2025. We included adults (≥ 18 years) with ischaemic stroke (ICD-10: I63) who underwent EVT within 24 h of stroke onset (identified via CPT codes; see Supplementary Table 1). Only patients with serum albumin measurements available within 24 h post-EVT were included to assess the prognostic role of albumin in acute phase. The study period was from 1 September 2018 to 1 September 2024. Given the cohorts were predominantly derived from the United States, the start date of 1 September 2018 was chosen, because the American Heart Association/American Stroke Association guideline for early management of patients with acute ischaemic stroke was originally published in January 2018^14^, while 1 September 2024 was selected to ensure that all included patients had completed the minimum follow-up period at the time of data analysis. The baseline index event was the date of the reported ischaemic stroke diagnosis; any diagnoses recorded prior to this date were regarded as the baseline characteristics of the participant. To ensure that baseline covariates were captured before the ischaemic stroke, we applied a one-day look-back window, including only variables recorded up to one day before the index event.

Patients were stratified into two groups based on albumin levels within 24 h post-stroke: normal (≥ 3.5 g/dL) and reduced (≤ 3.4 g/dL), based on thresholds established in previous studies demonstrating hypoalbuminemia to early cardiovascular events and mortality in patients with ischaemic stroke^9,10^, as well as an increased one-year risk of cardiovascular events in acutely ill medical patients^15^. Stroke severity was assessed using the National Institutes of Health Stroke Scale (NIHSS), a validated 42-point tool where scores > 16 are associated with higher mortality^16^.

### Outcomes

The primary outcome was a composite of all-cause mortality, major cardiovascular events within 30 days, including acute myocardial infarction (I21), acute heart failure (I50 subcodes), Takotsubo syndrome (I51.81), atrial fibrillation/flutter (I48), and ventricular arrythmias (I47.2, I49.0). Secondary outcomes included each component of the composite and intracranial haemorrhage (I60–I62). Outcomes were assessed from day 1 to day 30 post-stroke. To reduce confounding from pre-existing conditions, patients with a documented history of the outcomes (e.g., prior atrial fibrillation or heart failure) before the stroke were excluded. To test the consistency of the analysis, we evaluated the most common physiological and pathological conditions associated with reduced albumin levels through three subgroup analyses and three sensitivity analyses. The first subgroup analysis included patients aged ≥ 65 years, in whom hypoalbuminemia is a known mortality risk factor, whether they live in the community or they are in hospital or institutionalized^17^. Second subgroup analysis included female patients that have been shown to be more vulnerable to post-stroke cardiac complications^18^. In the third subgroup analysis, patients were stratified by stroke severity into mild/moderate (NIHSS 0–15) and severe (NIHSS 16–42) categories. Stroke severity was selected as a stratification variable because more severe strokes are associated with greater autonomic dysregulation^19^, and an increased risk of post-stroke cardiac complications falling within the SHS spectrum^20^. The first sensitivity analyses excluded patients with conditions influencing albumin (malnutrition or other plasma-protein metabolism disorder, glomerular (including nephrotic and nephritic syndromes) and liver diseases. The second sensitivity analysis stratifying hypoalbuminemia into: mild (2.7–3.5 g/dL) and severely (≤ 2.7 g/dL), as previously described^9^. To assess consistency with prior literature, we conducted an exploratory analysis of a parallel cohort of non-EVT ischaemic stroke patients stratified by albumin levels. This analysis was intended to examine whether similar albumin–outcome associations were observed in a broader stroke population.

### Statistical analysis

All analyses were conducted on the TriNetX platform. Baseline characteristics were compared using χ^2^ tests for categorical and independent-sample t-tests for continuous variables.

To adjust for confounders, 1:1 propensity score matching (PSM) was performed using greedy nearest neighbour algorithm based on multiple covariates, including age, sex, ethnicity, hypertensive diseases, ischaemia heart disease, atrial fibrillation, heart failure, pulmonary heart disease and diseases of pulmonary circulation, disorders of lipoprotein metabolism and other dyslipidaemias, diabetes mellitus, obesity and other hyperalimentation, chronic kidney disease, cerebral infarction, other peripheral vascular diseases, symptoms and signs specifically associated with systemic inflammation and infection, systemic connective tissue disorders, malnutrition, nephrotic syndrome, cirrhosis of liver, ulcerative colitis, Crohn’s disease, burns and corrosions of external body surface, NIHSS score, echocardiography procedures, cardiac catheterization procedures, electrocardiogram, antilipemic agents, beta blockers, antiarrhythmics, diuretics, calcium channel blockers, ACE inhibitors, angiotensin II inhibitors, antianginals, anticoagulants, platelet aggregation inhibitors, alteplase, and Tenecteplase. Covariates with an absolute standardised mean < 0.1 were considered well-matched.

Differences in cumulative risk for the composite outcome between the matched cohorts were assessed using log-rank tests and visualised with Kaplan–Meier curves. Cox proportional hazards regression analysis estimated hazard ratios (HRs) with 95% confidence intervals (CI) for the risk of adverse events between the cohorts. The proportional hazards assumption was tested using scaled Schoenfeld residuals. All tests were two-tailed with P ≤ 0.05 considered statistically significant. Analyses used the R survival package v3.2–3, integrated in the TriNetX platform.

### Ethics approval and consent to participate

This study was a retrospective observational analysis conducted using the TriNetX LIVE™ research network, which provides access to de-identified electronic health record data from healthcare organizations worldwide. The TriNetX platform complies with the Health Insurance Portability and Accountability Act (HIPAA) Privacy Rule (45 CFR §164.514[b][1]) and contains only de-identified patient data. Approval from the University of Liverpool institutional Review Board was not required for this study due to the exclusive use of de-identified data and absence of direct interaction with human subjects. Informed consent was not required. The study was conducted in accordance with applicable guidelines and regulations, including the Declaration of Helsinki, and is supported in accordance with the STROBE reporting guidelines.

## Results

**Fig. 1:**
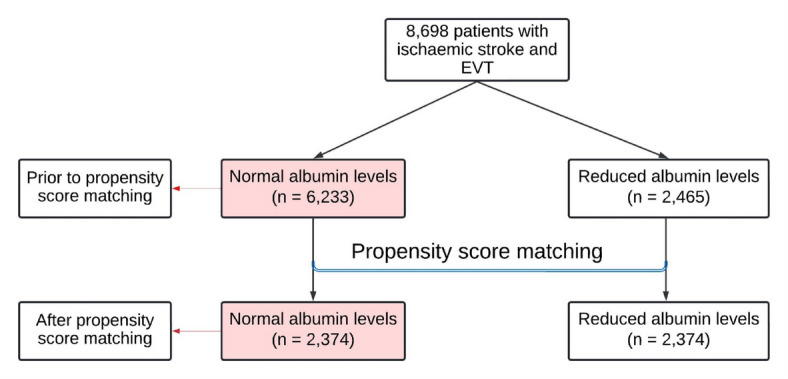
Flow diagram of selected patients with ischaemic stroke and albumin levels. EVT, endovascular thrombectomy.

The overall cohort included 8,698 patients, of whom 6,233 had normal serum albumin levels (mean age: 67.0 ± 14.5 years, 44.0% female) and 2,465 had reduced albumin levels (mean age: 69.7 ± 14.3 years, 51.5% female, Fig. 1). Before PSM, patients with reduced albumin levels exhibited a higher prevalence of cerebral infarction, cardiovascular disease, chronic kidney disease, liver cirrhosis, and were more likely to be prescribed anticoagulants and antiplatelet (Table 1). After PSM, 2,374 patients were included in each group, with balanced baseline characteristics (all absolute standardised difference < 0.1; Table 1).

**Table 1:** Baseline characteristics of patients with stroke with reduced and normal albumin levels before and after propensity score matching.

	Before propensity score matching			After propensity score matching		
	Reduced albumin levels N = 2,465	Normal albumin levels N = 6,233	ASD	Reduced albumin level N = 2,374	Normal albumin level N = 2,374	ASD
Age, y (± SD)	69.7 ± 14.3	67.0 ± 14.5	0.193	69.4 ± 14.3	69.8 ± 14.3	0.029
Female, n (%)	1,269 (51.5)	2,744 (44.0)	0.111	1,206 (50.8%)	1,232 (51.9)	0.022
White, n (%)	1,541 (62.5)	3,557 (57.1)	0.150	1,470 (61.9%)	1,471 (62.0%)	0.001
Black or African American, n (%)	381 (15.5)	894 (14.3)	0.031	366 (15.4%)	371 (15.6%)	0.006
Asian, n (%)	128 (5.2)	486 (7.8)	0.106	127 (5.3%)	126 (5.3%)	0.002
Hypertension, n (%)	725 (29.4)	1,308 (21.0)	0.195	654 (27.5%)	611 (25.7%)	0.041
Ischaemic heart disease, n (%)	285 (11.6)	409 (6.6)	0.175	233 (9.8%)	216 (9.1%)	0.024
Atrial fibrillation, n (%)	396 (16.1)	563 (9.0)	0.213	332 (14.0%)	327 (13.8%)	0.006
Heart failure, n (%)	242 (9.8)	292 (4.7)	0.199	194 (8.2%)	180 (7.6%)	0.022
Pulmonary heart disease, n (%)	96 (3.9)	120 (1.9)	0.117	78 (3.3%)	77 (3.2%)	0.002
Lipoprotein disorder, n (%)	481 (19.5)	900 (14.4)	0.135	445 (18.7%)	400 (16.8%)	0.050
Diabetes mellitus, n (%)	267 (10.8)	424 (6.8)	0.142	238 (10.0%)	205 (8.6%)	0.048
Obesity, n (%)	153 (6.2)	312 (5.0)	0.052	145 (6.1%)	125 (5.3%)	0.036
Chronic kidney disease, n (%)	138 (5.6)	160 (2.6)	0.154	109 (4.6%)	109 (4.6%)	< 0.001
Cerebral infarction, n (%)	821 (33.3)	1,296 (20.8)	0.285	733 (30.9%)	690 (29.1%)	0.040
Peripheral vascular disease, n (%)	54 (2.2)	81 (1.3)	0.068	48 (2.0%)	44 (1.9%)	0.012
Symptoms and signs associated with systemic inflammation and infection, n (%)	34 (1.4)	27 (0.4)	0.100	21 (0.9%)	21 (0.9)	< 0.001
Systemic connective tissue disorder (%)	11 (0.4)	28 (0.4)	< 0.001	10 (0.4%)	10 (0.4%)	< 0.001
Malnutrition, n (%)	63 (2.6)	31 (0.5)	0.168	31 (1.3%)	29 (1.2%)	0.008
Nephrotic syndrome, n (%)	0	10 (0.2)	0.057	0	0	–
Cirrhosis of liver, n (%)	10 (0.4)	10 (0.2)	0.046	10 (0.4%)	10 (0.4%)	< 0.001
Ulcerative colitis, n (%)	10 (0.4)	10 (0.2)	0.046	10 (0.4%)	10 (0.4%)	< 0.001
Crohn’s disease, n (%)	10 (0.4)	10 (0.2)	0.046	10 (0.4%)	10 (0.4%)	< 0.001
Burns and corrosions of external body surface, n (%)	10 (0.4)	10 (0.2)	0.046	10 (0.4%)	10 (0.4%)	< 0.001
NIHSS, n (%)	382 (15.5)	643 (10.3)	0.155	342 (14.4%)	319 (13.4%)	0.028
Echocardiography Procedures, n (%)	161 (6.5)	223 (3.6)	0.135	137 (5.8%)	128 (5.4%)	0.017
Cardiac Catheterization Procedures, n (%)	19 (0.8)	26 (0.4)	0.046	17 (0.7%)	14 (0.6%)	0.016
Electrocardiogram, routine ECG with at least 12 leads, n (%)	511 (20.7)	864 (13.9)	0.182	463 (19.5%)	432 (18.2%)	0.033
Antilipemic agents, n (%)	438 (17.8)	781 (12.5)	0.147	396 (16.7%)	368 (15.5%)	0.032
Beta blockers/related, n (%)	601 (24.4)	945 (15.2)	0.233	538 (22.7%)	503 (21.2%)	0.036
Antiarrhythmics, n (%)	523 (21.2)	796 (12.8)	0.226	465 (19.6%)	431 (18.2%)	0.037
Diuretics, n (%)	210 (8.5)	407 (6.5)	0.075	192 (8.1%)	183 (7.7%)	0.014
Calcium channel blockers, n (%)	435 (17.6)	734 (11.8)	0.166	389 (16.4%)	353 (14.9%)	0.042
Ace inhibitors, n (%)	175 (7.1)	330 (5.3)	0.075	161 (6.8%)	137 (5.8%)	0.042
Angiotensin II inhibitor, n (%)	134 (5.4)	249 (4.0)	0.068	122 (5.1%)	117 (4.9%)	0.010
Antianginals, n (%)	121 (4.9)	212 (3.4)	0.076	105 (4.4%)	93 (3.9%)	0.025
Anticoagulants, n (%)	516 (20.9)	712 (11.4)	0.260	450 (19.0%)	434 (18.3%)	0.017
Platelet aggregation inhibitors, n (%)	325 (13.2)	597 (9.6)	0.114	297 (12.5%)	262 (11.0%)	0.046
Alteplase, n (%)	109 (4.4)	188 (3.0)	0.074	103 (4.3%)	93 (3.9%)	0.021
Tenecteplase, n (%)	46 (1.9)	85 (1.4)	0.040	42 (1.8%)	37 (1.6%)	0.016

**Fig. 2:**
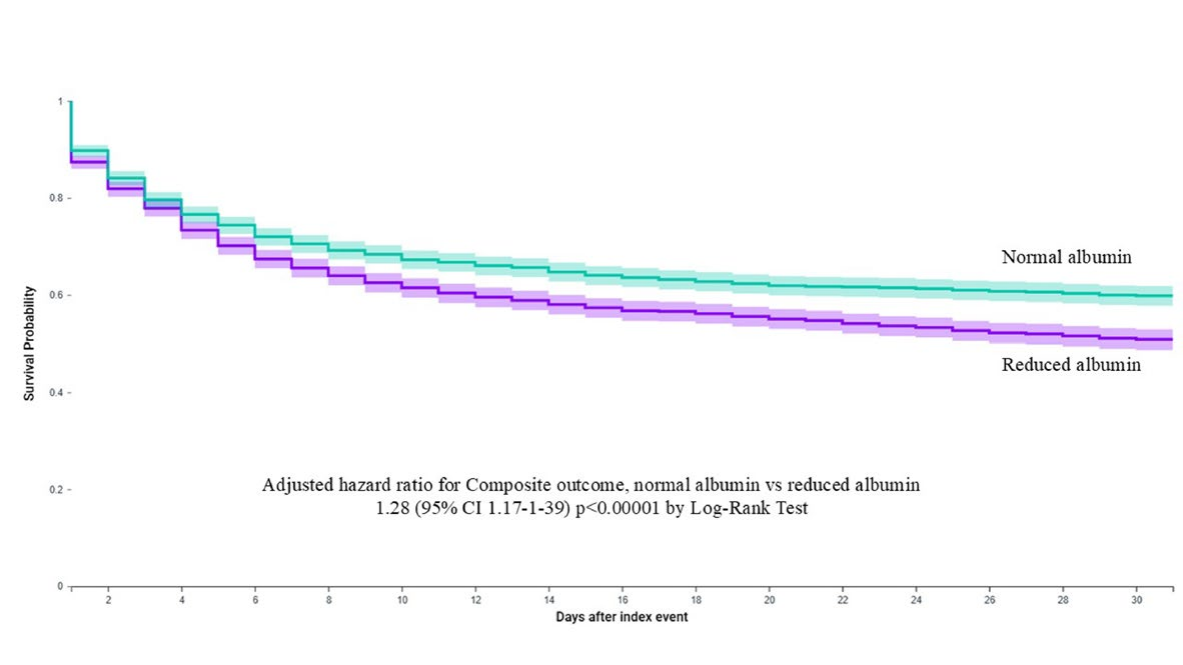
Kaplan–Meier curve for the composite outcome within 30 days from stroke onset in patients with normal (green) and reduced (purple) albumin levels.

**Fig. 3:**
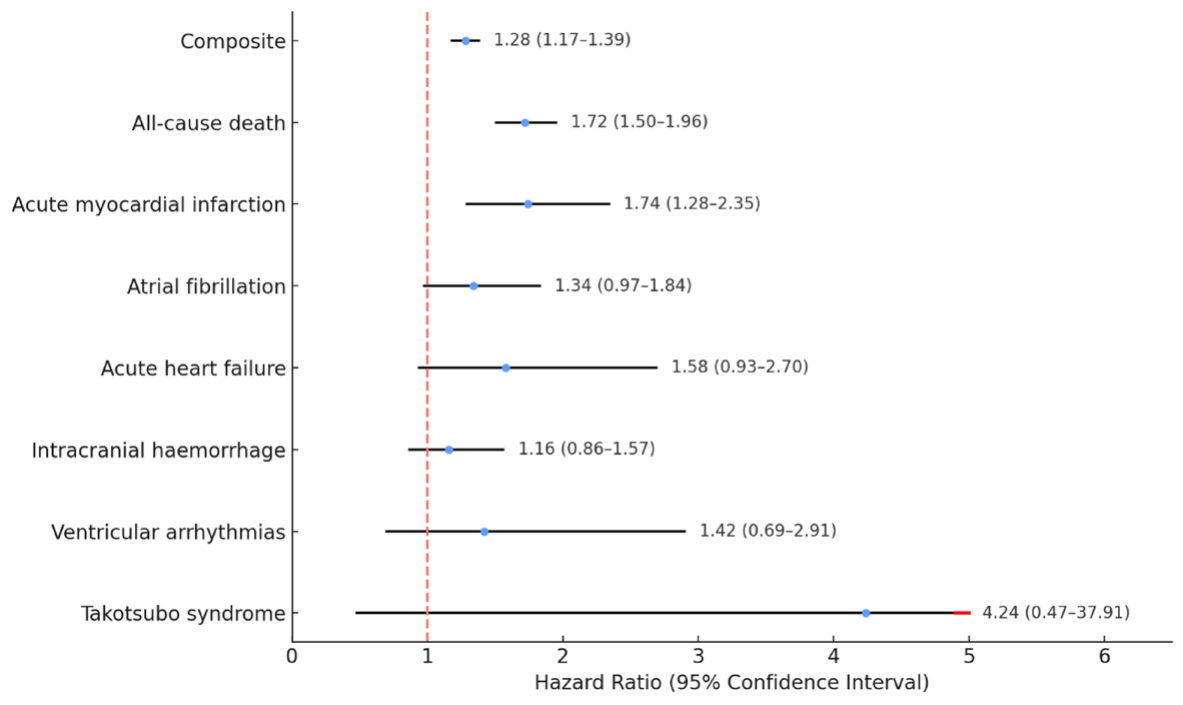
Forest Plot illustrating the thirty-day risk of early cardiovascular complications following stroke in patients with normal versus reduced serum albumin levels. HR denotes Hazard Ratio. The red dashed line signifies that the confidence interval exceeds the bounds of the graph displayed in the figure.

Cox regression analysis performed on the matched cohort showed that, within 30 days of the acute ischaemic stroke, patients with reduced albumin levels had a significantly higher risk of the composite outcome of cardiovascular events or death (HR 1.28, 95%CI 1.17–1.39), all-cause mortality (HR 1.72, 95%CI 1.50–1.96), and acute myocardial infarction (HR 1.74, 95%CI 1.28–2.35) (Fig. 2). However, there were no statistically significant differences between the groups in the risk of acute heart failure (HR 1.58, 95%CI 0.93–2.70), Takotsubo syndrome (HR 4.24, 95%CI 0.47–37.91), atrial fibrillation (HR 1.34, 95%CI 0.97–1.84), ventricular arrhythmias (HR 1.42, 95%CI 0.69–2.91), or intracranial haemorrhage (HR 1.16, 95%CI 0.86–1.57) (Fig. 3).

### Subgroup analyses

#### Older age (≥ 65)

**Fig. 4:**
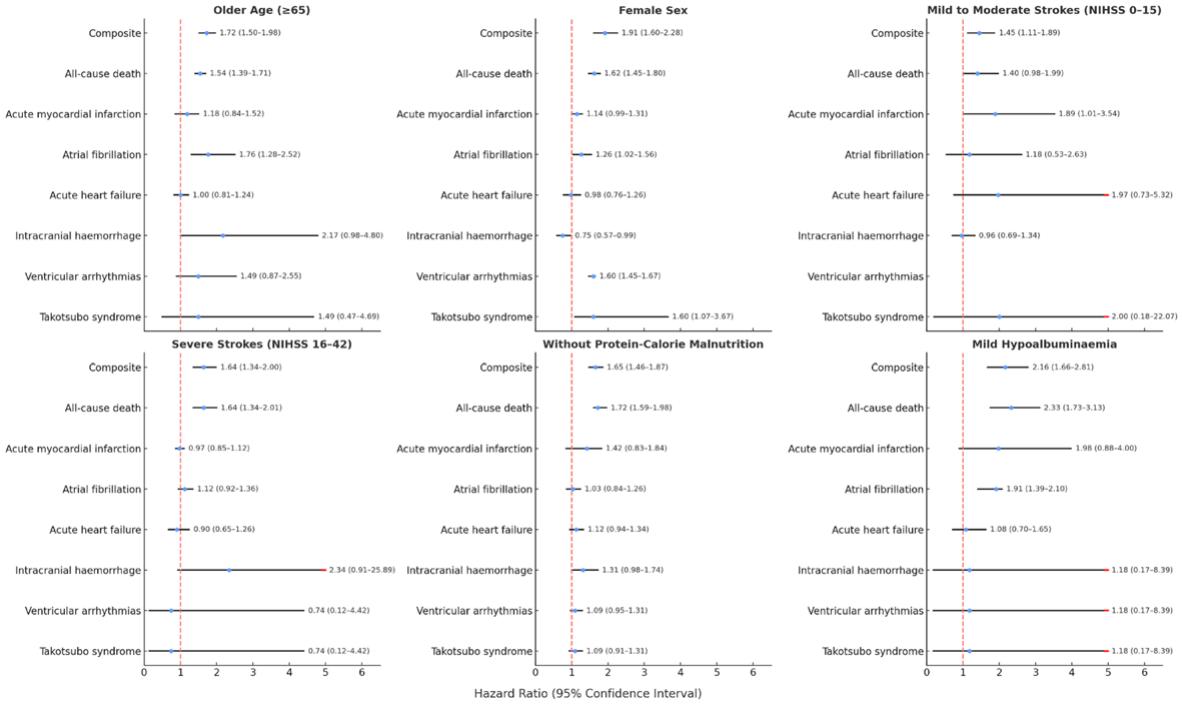
Forest Plot illustrating the thirty-day risk of early cardiovascular complications following stroke across different subgroups. HR denotes Hazard Ratio. The red dashed line signifies that the confidence interval exceeds the bounds of the graph displayed in the figure.

After PSM, each cohort included 1,593 patients aged > 65 years (Supplementary Table 2). Those with reduced albumin levels showed a higher risk of the composite outcome (HR 1.72, 95%CI 1.50–1.98), all-cause mortality (HR 1.77, 95%CI 1.51–2.07), and acute myocardial infarction (HR 2.32, 95%CI 1.52–3.55). No statistically significant differences were observed for the risks of acute heart failure (HR 1.76, 95%CI 0.88–3.52), Takotsubo syndrome (HR 1.49, 95%CI: 0.47–4.69), atrial fibrillation (HR 1.18 95%CI 0.84–1.65), ventricular arrhythmias (HR 2.55, 95%CI 0.98–6.64), or intracranial haemorrhage (HR 1.00, 95%CI 0.81–1.22) in this age group (Fig. 4).

#### Female sex

Among female patients, each matched cohort consisted of 1,197 individuals (Supplementary Table 3). Those with reduced albumin levels had a significantly higher risk of the composite outcome (HR 1.91, 95%CI 1.60–2.28) and all-cause mortality (HR 2.04, 95%CI 1.67–2.50). There were no significant differences in the risk of acute myocardial infarction (HR 1.61, 95%CI 0.99–2.61), acute heart failure (HR 1.23, 95%CI 0.54–2.78), Takotsubo syndrome (HR 1.60, 95%CI 0.45–5.67), atrial fibrillation (HR 1.12, 95%CI 0.72–1.73), ventricular arrhythmias (HR 0.75, 95%CI 0.21–2.65), or intracranial haemorrhage (HR 0.98, 95%CI 0.76–1.26) (Fig. 4).

#### Mild to moderate strokes

In patients with mild to moderate stroke severity (NIHSS score 0–15), each group included 642 matched individuals (Supplementary Table 4). Those with reduced albumin levels had an increased risk of the composite outcome (HR 1.45, 95%CI 1.11–1.89) and acute myocardial infarction (HR 1.89, 95%CI 1.01–3.54). No significant differences were found for the risk of all-cause mortality (HR 1.40, 95%CI 0.98–1.99), acute heart failure (HR 1.97, 95%CI 0.73–5.32), Takotsubo syndrome (HR 2.00, 95%CI 0.18–22.07), atrial fibrillation (HR 1.18, 95%CI 0.53–2.63), or intracranial haemorrhage (HR 0.96, 95%CI 0.69–1.34). No cases of ventricular arrhythmia were reported in patients with reduced albumin levels (Fig. 4).

#### Severe strokes

In patients with severe stroke (NIHSS score 16–42), each matched group included 664 individuals (Supplementary Table 5). Those with reduced albumin levels had a higher risk of the composite outcome (HR 1.64, 95%CI 1.34–2.00) and all-cause mortality (HR 1.64, 95%CI 1.31–2.04). No significant differences were found in the risk of acute myocardial infarction (HR 1.22, 95%CI 0.69–2.16), acute heart failure (HR 0.95, 95%CI 0.26–3.54), Takotsubo syndrome (HR 0.74, 95%CI 0.12–4.42), atrial fibrillation (HR 0.97, 95%CI 0.35–2.68), ventricular arrhythmias (HR 2.34, 95%CI 0.21–25.89), or intracranial haemorrhage (HR 0.90, 95%CI 0.65–1.25) (Fig. 4).

### Sensitivity analysis

#### Patients without protein-calorie malnutrition or other plasma-protein metabolism disorder and glomerular diseases and liver diseases

After PSM, 2,158 patients without protein-calorie malnutrition or other plasma-protein metabolism disorder and glomerular disease and liver disease remained in each group (Supplementary Table 6)*.* Those with reduced albumin levels had a significantly higher risk of the composite outcome (HR 1.65, 95%CI 1.46–1.87), all-cause mortality (HR 1.72, 95%CI 1.50–1.98), and acute myocardial infarction (HR 1.81, 95%CI 1.30–2.51). No significant differences were observed in the risks of acute heart failure (HR 1.42, 95%CI 0.83–2.42), Takotsubo syndrome (HR 1.90, 95%CI 0.64–5.68), atrial fibrillation (HR 1.31, 95%CI 0.93–1.84), ventricular arrhythmias (HR 1.09, 95%CI 0.54–2.17), or intracranial haemorrhage (HR 1.09, 95%CI 0.91–1.31) (Fig. 4).

#### Mild hypoalbuminaemia

Further stratification of reduced albumin levels showed that, among patients with mild hypoalbuminaemia (2.7–3.4 g/dL), each cohort included 2,302 patients after matching (Supplementary Table 7). This group had a higher risk of the composite outcome (HR 1.53, 95%CI 1.36–1.73), all-cause mortality (HR 1.57, 95%CI 1.36–1.81), and acute myocardial infarction (HR 1.90, 95%CI 1.38–2.60). No significant differences were found for the risk of acute heart failure (HR 1.28, 95%CI 0.75–2.20), Takotsubo syndrome (HR 1.66, 95%CI 0.54–5.07), atrial fibrillation (HR 0.99, 95%CI 0.71–1.37), ventricular arrhythmias (HR 1.06, 95%CI 0.53–2.11), or intracranial haemorrhage (HR 1.05, 95%CI 0.88–1.25) (Fig. 4).

#### Severe hypoalbuminaemia

Among patients with severe hypoalbuminemia (≤ 2.7 g/dL), each matched group included 402 individuals (Supplementary Table 8). This group exhibited significantly higher risks of the composite outcome (HR 2.16, 95%CI 1.66–2.81) and all-cause mortality (HR 2.33, 95%CI 1.73–3.13). No significant differences were found for the risk of acute myocardial infarction (HR 1.98, 95%CI 0.98–4.00), acute heart failure (HR 9.30, 95%CI 1.16–74.46), atrial fibrillation (HR 0.91, 95%CI 0.39–2.10), ventricular arrhythmias (HR 1.18, 95%CI 0.17–8.39), or intracranial haemorrhage (HR 1.08, 95%CI 0.70–1.65). No cases of Takotsubo syndrome were recorded in patients with normal albumin levels (Fig. 4).

#### Non-EVT ischaemic stroke patients

After PSM, each cohort included 36,498 patients with AIS who did not undergo EVT (Supplementary Table 9). Those with reduced albumin levels had a significantly higher risk of the composite outcome (HR 1.95, 95%CI 1.90–2.01), all-cause mortality (HR 3.29, 95%CI 3.12–3.46), acute myocardial infarction (HR 2.19, 95%CI 2.04–2.35), acute heart failure (HR 2.22, 95%CI 1.86–2.66), Takotsubo syndrome (HR 2.51, 95%CI 1.35–4.68), atrial fibrillation (HR 1.35, 95%CI 1.22–1.49), ventricular arrhythmias (HR 1.48, 95%CI 1.21–1.82), or intracranial haemorrhage (HR 1.54, 95%CI 1.37–1.73).

## Discussion

This study found that patients with serum albumin levels ≤ 3.4 g/dL, measured within 24 h of an acute ischaemic stroke and treated with EVT, had a significantly higher risk of composite cardiovascular outcomes, all-cause mortality, and acute myocardial infarction within 30 days, compared to those with albumin levels ≥ 3.5 g/dL. These associations remained consistent across all pre-specified subgroups, including older adults (≥ 65 years), female, and patients with varying stroke severity. Importantly, both mild and severe hypoalbuminaemia were independently associated with increased risk of adverse outcomes, even after excluding patients with conditions commonly linked to chronic hypoalbuminaemia, such as malnutrition, plasma protein metabolism disorders, glomerular diseases, and liver disease.

While cardiovascular complications after stroke are well recognised^1–4^, whether EVT contributes to this risk remains unclear. Emerging evidence suggests that EVT may exacerbate systemic endothelial injury due to direct manipulation of the arterial wall^21,22^. This is particularly relevant given that EVT is typically performed in cases of large vessel occlusion^14^, which is inherently associated with poorer outcomes compared to strokes without large vessel involvement^23^. The mechanical and inflammatory stress from EVT may compound the oxidative and inflammatory damage already triggered by the stroke^24^, potentially extending to coronary microvasculature or epicardial vessels and increasing the risk of myocardial injury, arrhythmias, and heart failure^25–28^.

Endothelial injury and inflammation are closely linked processes that amplify one another, potentially leading to organ damage in the absence of protective factors. Albumin plays a key protective role in this context by modulating oxidative stress and inflammation. It scavenges reactive oxygen species^8,29^ and inhibits monocyte adhesion to vascular endothelium^6^, thereby reducing the risk of further vascular damage. Beyond its anti-inflammatory effects, albumin also has anticoagulant and antiplatelet properties. It inhibits histone-induced platelet aggregation^30^, binds thromboxane A2^31^, and enhances antithrombin III activity^32^, all of which contribute to maintaining vascular homeostasis. Reduced albumin levels may impair these protective mechanisms and increase susceptibility to thrombotic events.

As a negative acute-phase reactant, albumin levels typically decrease during systemic inflammation. Although hepatic synthesis may increase during acute phase, consumption and degradation are accelerated, and albumin is used as an intracellular amino acid donor in stressed tissues^33^. Thus, hypoalbuminaemia can reflect both the severity of systemic inflammation and underlying poor physiological reserve. In this context, reduced albumin levels may serve as a marker of poor underlying health status that are independently associated with poor prognosis, regardless of any direct mechanistic role in driving adverse outcomes^34^.

Findings in the parallel non-EVT cohort showed consistent directionality of albumin–outcome associations, supporting the robustness of our results and alignment with previous studies linking hypoalbuminemia to poor outcomes in stroke patients^9,15^. In the “REgistro POliterapie SIMI” (REPOSI) study, involving 4,152 patients, those with albumin ≤ 3.4 g/dL (47.2%) had significantly higher risks of all-cause mortality and ischemic events at 12 months^15^. Similarly, a retrospective observational study of 329,734 stroke patients showed significantly increased risks of all-cause mortality and cardiovascular complications within 30 days of stroke among those with low albumin^9^. Compared to that study, our findings, focused specifically on EVT-treated patients, identified a narrow range of statistically significant outcomes, namely, the composite outcomes, all-cause mortality, and acute myocardial infarction. Although atrial fibrillation and heart failure did not reach statistical significance in our analysis, they showed a trend toward increased risk in the hypoalbuminaemia group. The absence of statistical significance for other cardiovascular complications in our cohort may reflect the smaller sample size and the specific characteristics of EVT patients, who represent a distinct, high-risk subgroup.

Whether correcting hypoalbuminaemia can improve outcomes remains uncertain. Interventional studies in critically ill patients showed mixed results^35–38^. However, given the biological roles of albumin and its strong prognostic value, routine measurement may support early risk stratification in stroke patient undergoing EVT. Its low cost, broad availability, and biological plausibility make serum albumin an attractive candidate biomarker for clinical decision-making.

Compared to other established predictors such as age, stroke severity (NIHSS), and inflammatory markers, albumin may provide complementary prognostic information without adding significant complexity or cost. While it may not independently influence EVT eligibility or immediate treatment decisions, serum albumin could still play a meaningful role within broader clinical risk stratification frameworks. Our findings therefore highlight the potential of albumin to complement existing tools, but also highlight the need for prospective studies to establish its true impact on clinical decision-making.

## Strengths and limitations

To our knowledge, this is the first study to assess cardiovascular outcomes in EVT-treated stroke patients stratified by serum albumin levels. Strengths include the large cohort size, use of real-world multicentre data, and rigorous confounder adjustment using PSM.

Nonetheless, several limitations warrant consideration. First, as a retrospective study, causality cannot be inferred, and residual confounding is possible. Second, data were drawn from an opt-in federated network, potentially introducing selection bias based on participating institutions. Third, use of electronic health records may lead to inconsistencies or missing data across centres. Fourth, residual confounding cannot be excluded, as several unmeasured factors may have influenced outcomes. These include functional status, dietary patterns, and socioeconomic conditions, as well as key determinants of stroke severity—such as infarct volume, vascular territory (anterior vs posterior circulation), collateral status, and quality of recanalisation—which were not available despite extensive propensity score matching. These factors may partially mediate the observed association between hypoalbuminaemia and adverse cardiovascular outcomes. Fifth, procedural details related to endovascular thrombectomy (device type, number of passes, procedure duration, periprocedural complications, recanalisation grade) were not available in the TriNetX database. These factors are known to strongly influence neurological and cardiovascular outcomes after EVT and may confound the relationship between albumin levels and post-stroke complications. Sixth, serum albumin was measured only once within a 24-h window, during which patients may have been at different stages of their clinical course (pre-procedural, immediately post-EVT, or during early intensive care management). Albumin concentrations are sensitive to haemodilution, fluid resuscitation, and early inflammatory changes, which may have introduced measurement variability and potential non-differential misclassification. Consequently, albumin levels in this study should be interpreted as an early prognostic snapshot rather than a stable biomarker over time. Seventh, analyses were limited to patients with available albumin values. This introduces potential selection bias, as patients undergoing laboratory testing may differ systematically from those without testing. As a result, the findings are most generalizable to EVT patients who had albumin measured, rather than to the entire EVT population. Eight, the composite outcome used in this study reflects the clinical construct of SHS and aligns with definitions used in prior literature^9^. However, it combines heterogeneous cardiovascular events of varying severity and diagnostic certainty. While this definition captures the multifaceted nature of SHS, it may reduce interpretability. Despite these limitations, the study’s sample size and multicentre scope enhance the generalisability and robustness of its findings.

## Conclusion

In patients with acute ischaemic stroke treated with EVT, hypoalbuminaemia was associated with a high risk of death and major cardiovascular events. Serum albumin may serve as a useful and accessible biomarker for identifying patients at higher risk following EVT. Further prospective studies are needed to determine whether therapeutic correction of hypoalbuminaemia can improve clinical outcomes in this high-risk population.

## Supplementary Information

Below is the link to the electronic supplementary material.

Supplementary Material 1

## Data Availability

The data underlying this article are available in the TriNetX research network at https://live.trinetx.com with a request for access to the TriNetX network, but costs may be incurred.
