# Supplementary material for: Albumin levels and risk of early cardiovascular complications after endovascular thrombectomy for acute ischaemic stroke
Source: Sci Rep. 2026 Apr 24;16:18924. doi: 10.1038/s41598-026-50280-0 (PMC13276201; doi:10.1038/s41598-026-50280-0)
Supplement: Supplementary file 1 — Supplementary Material 1 [file 41598_2026_50280_MOESM1_ESM.docx]

**Supplementary Table 1**. ICD-10-CM or CPT codes for patients with acute ischaemic stroke treated by EVT.

| ICD-10 and CPT codes | |
| --- | --- |
| ICD-10 code | Description |
| I21 | Acute myocardial infarction |
| 12.0 | [ST elevation (STEMI) myocardial infarction of anterior wall](https://www.aapc.com/codes/icd-10-codes/I21.0) |
| 121.1 | [ST elevation (STEMI) myocardial infarction of inferior wall](https://www.aapc.com/codes/icd-10-codes/I21.1) |
| 121.2 | [ST elevation (STEMI) myocardial infarction of other sites](https://www.aapc.com/codes/icd-10-codes/I21.2) |
| 121.3 | [ST elevation (STEMI) myocardial infarction of unspecified site](https://www.aapc.com/codes/icd-10-codes/I21.3) |
| 121.4 | [Non-ST elevation (NSTEMI) myocardial infarction](https://www.aapc.com/codes/icd-10-codes/I21.4) |
| 121.9 | [Acute myocardial infarction, unspecified](https://www.aapc.com/codes/icd-10-codes/I21.9) |
| 121.A | [Other type of myocardial infarction](https://www.aapc.com/codes/icd-10-codes/I21.A) |
| 121.B | [Myocardial infarction with coronary microvascular dysfunction](https://www.aapc.com/codes/icd-10-codes/I21.B) |
| [I47.2](https://www.aapc.com/codes/icd-10-codes/I47.2) | Ventricular tachycardia |
| I48 | Atrial fibrillation and flutter |
| [I48.0](https://www.aapc.com/codes/icd-10-codes/I48.0) | [Paroxysmal atrial fibrillation](https://www.aapc.com/codes/icd-10-codes/I48.0) |
| [I48.1](https://www.aapc.com/codes/icd-10-codes/I48.1) | [Persistent atrial fibrillation](https://www.aapc.com/codes/icd-10-codes/I48.1) |
| [I48.2](https://www.aapc.com/codes/icd-10-codes/I48.2) | [Chronic atrial fibrillation](https://www.aapc.com/codes/icd-10-codes/I48.2) |
| [I48.3](https://www.aapc.com/codes/icd-10-codes/I48.3) | [Typical atrial flutter](https://www.aapc.com/codes/icd-10-codes/I48.3) |
| [I48.4](https://www.aapc.com/codes/icd-10-codes/I48.4) | [Atypical atrial flutter](https://www.aapc.com/codes/icd-10-codes/I48.4) |
| [I48.9](https://www.aapc.com/codes/icd-10-codes/I48.9) | [Unspecified atrial fibrillation and atrial flutter](https://www.aapc.com/codes/icd-10-codes/I48.9) |
| [I49](javascript:void();) | [Other cardiac arrhythmias](javascript:void();) |
| [I49.0](https://www.aapc.com/codes/icd-10-codes/I49.0) | [Ventricular fibrillation and flutter](https://www.aapc.com/codes/icd-10-codes/I49.0) |
| [I49.1](https://www.aapc.com/codes/icd-10-codes/I49.1) | [Atrial premature depolarization](https://www.aapc.com/codes/icd-10-codes/I49.1) |
| [I49.2](https://www.aapc.com/codes/icd-10-codes/I49.2) | [Junctional premature depolarization](https://www.aapc.com/codes/icd-10-codes/I49.2) |
| [I49.3](https://www.aapc.com/codes/icd-10-codes/I49.3) | [Ventricular premature depolarization](https://www.aapc.com/codes/icd-10-codes/I49.3) |
| [I49.4](https://www.aapc.com/codes/icd-10-codes/I49.4) | [Other and unspecified premature depolarization](https://www.aapc.com/codes/icd-10-codes/I49.4) |
| [I49.5](https://www.aapc.com/codes/icd-10-codes/I49.5) | [Sick sinus syndrome](https://www.aapc.com/codes/icd-10-codes/I49.5) |
| [I49.8](https://www.aapc.com/codes/icd-10-codes/I49.8) | [Other specified cardiac arrhythmias](https://www.aapc.com/codes/icd-10-codes/I49.8) |
| [I49.9](https://www.aapc.com/codes/icd-10-codes/I49.9) | [Cardiac arrhythmia, unspecified](https://www.aapc.com/codes/icd-10-codes/I49.9) |
| I50.21 | [Acute systolic (congestive) heart failure](https://www.aapc.com/codes/icd-10-codes/I50.21) |
| I50.23 | Acute on chronic systolic (congestive) heart failure |
| I50.31 | [Acute diastolic (congestive) heart failure](https://www.aapc.com/codes/icd-10-codes/I50.31) |
| [I50.41](https://www.aapc.com/codes/icd-10-codes/I50.41) | [Acute combined systolic (congestive) and diastolic (congestive) heart failure](https://www.aapc.com/codes/icd-10-codes/I50.41) |
| I50.43 | [Acute on chronic combined systolic (congestive) and diastolic (congestive) heart failure](https://www.aapc.com/codes/icd-10-codes/I50.43) |
| I51.81 | [Takotsubo syndrome](https://www.aapc.com/codes/icd-10-codes/I51.81) |
| I60 | Nontraumatic subarachnoid hemorrhage |
| 160.1 | Nontraumatic subarachnoid hemorrhage from middle cerebral |
| 160.2 | Nontraumatic subarachnoid hemorrhage from anterior communicating artery |
| 160.3 | Nontraumatic subarachnoid hemorrhage from posterior communicating artery |
| 160.4 | Nontraumatic subarachnoid hemorrhage from basilar artery |
| 160.5 | Nontraumatic subarachnoid hemorrhage from vertebral artery |
| I60.6 | Nontraumatic subarachnoid hemorrhage from other intracranial arteries |
| I60.7 | Nontraumatic subarachnoid hemorrhage from unspecified intracranial artery |
| I60.8 | Other nontraumatic subarachnoid hemorrhage |
| I60.9 | [Nontraumatic subarachnoid hemorrhage, unspecified](https://www.aapc.com/codes/icd-10-codes/I60.9) |
| I61 | Nontraumatic intracerebral hemorrhage |
| I61.0 | Nontraumatic intracerebral hemorrhage in hemisphere, subcortical |
| I61.1 | [Nontraumatic intracerebral hemorrhage in hemisphere, cortical](https://www.aapc.com/codes/icd-10-codes/I61.1) |
| I61.2 | Nontraumatic intracerebral hemorrhage in hemisphere, unspecified |
| I61.3 | Nontraumatic intracerebral hemorrhage in brain stem |
| I61.4 | Nontraumatic intracerebral hemorrhage in cerebellum |
| I61.5 | Nontraumatic intracerebral hemorrhage, intraventricular |
| I61.6 | Nontraumatic intracerebral hemorrhage, multiple localized |
| I61.8 | Other nontraumatic intracerebral hemorrhage |
| I61.9 | Nontraumatic intracerebral hemorrhage, unspecified |
| I62 | Other and unspecified nontraumatic intracranial hemorrhage |
| [I62.0](https://www.aapc.com/codes/icd-10-codes/I62.0) | Nontraumatic subdural hemorrhage |
| I62.1 | [Nontraumatic extradural hemorrhage](https://www.aapc.com/codes/icd-10-codes/I62.1) |
| I62.9 | [Nontraumatic intracranial hemorrhage, unspecified](https://www.aapc.com/codes/icd-10-codes/I62.9) |
| I63.52  I63.53 | Cerebral infarction (thrombosis & embolism & occlusion or stenosis of precerebral, anterior, middle, carotid, basilar and vertebral) |
| BL110 | Anticoagulants |
| 259280 | Tenecteplase |
| 8410 | Alteplase |
| ICD-10 code | Stroke severity |
| R29.7 | National Institutes of Health Stroke Scale (NIHSS) score |
| CPT | Mechanical Endovascular Reperfusion Procedures |
| 03CG3Z7 | Extirpation of Matter from Intracranial Artery using Stent Retriever, Percutaneous Approach |
| 03CG3ZZ | Extirpation of Matter from Intracranial Artery, Percutaneous Approach |
| 03CG4ZZ | Extirpation of Matter from Intracranial Artery, Percutaneous Endoscopic Approach |
| 03CH3Z7 | Extirpation of Matter from Right Common Carotid Artery using Stent Retriever, Percutaneous Approach |
| 03CH3ZZ | Extirpation of Matter from Right Common Carotid Artery, Percutaneous Approach |
| 03CH4ZZ | Extirpation of Matter from Right Common Carotid Artery, Percutaneous Endoscopic Approach |
| 03CJ3Z7 | Extirpation of Matter from Left Common Carotid Artery using Stent Retriever, Percutaneous Approach |
| 03CJ3ZZ | Extirpation of Matter from Left Common Carotid Artery, Percutaneous Approach |
| 03CJ4ZZ | Extirpation of Matter from Left Common Carotid Artery, Percutaneous Endoscopic Approach |
| 03CK3Z7 | Extirpation of Matter from Right Internal Carotid Artery using Stent Retriever, Percutaneous Approach |
| 03CK3ZZ | Extirpation of Matter from Right Internal Carotid Artery, Percutaneous Approach |
| 03CK4ZZ | Extirpation of Matter from Right Internal Carotid Artery, Percutaneous Endoscopic Approach |
| 03CL3Z7 | Extirpation of Matter from Left Internal Carotid Artery using Stent Retriever, Percutaneous Approach |
| 03CL3ZZ | Extirpation of Matter from Left Internal Carotid Artery, Percutaneous Approach |
| 03CL4ZZ | Extirpation of Matter from Left Internal Carotid Artery, Percutaneous Endoscopic Approach |
| 03CP3Z7 | Extirpation of Matter from Right Vertebral Artery using Stent Retriever, Percutaneous Approach |
| 03CP3ZZ | Extirpation of Matter from Right Vertebral Artery, Percutaneous Approach |
| 03CP4ZZ | Extirpation of Matter from Right Vertebral Artery, Percutaneous Endoscopic Approach |
| 03CQ3Z7 | Extirpation of Matter from Left Vertebral Artery using Stent Retriever, Percutaneous Approach |
| 03CQ3ZZ | Extirpation of Matter from Left Vertebral Artery, Percutaneous Approach |
| 03CQ4ZZ | Extirpation of Matter from Left Vertebral Artery, Percutaneous Endoscopic Approach |
| Thrombectomy Root Procedures | |
| 037G3ZZ | Dilation of Intracranial Artery, Percutaneous Approach |
| 037G4ZZ | Dilation of Intracranial Artery, Percutaneous Endoscopic Approach |
| 037H3ZZ | Dilation of Right Common Carotid Artery, Percutaneous Approach |
| 037H4ZZ | Dilation of Right Common Carotid Artery, Percutaneous Endoscopic Approach |
| 037J3ZZ | Dilation of Left Common Carotid Artery, Percutaneous Approach |
| 037J4ZZ | Dilation of Left Common Carotid Artery, Percutaneous Endoscopic Approach |
| 037K3ZZ | Dilation of Right Internal Carotid Artery, Percutaneous Approach |
| 037K4ZZ | Dilation of Right Internal Carotid Artery, Percutaneous Endoscopic Approach |
| 037L3ZZ | Dilation of Left Internal Carotid Artery, Percutaneous Approach |
| 037L4ZZ | Dilation of Left Internal Carotid Artery, Percutaneous Endoscopic Approach |
| 037P3ZZ | Dilation of Right Vertebral Artery, Percutaneous Approach |
| 037P4ZZ | Dilation of Right Vertebral Artery, Percutaneous Endoscopic Approach |
| 037Q3ZZ | Dilation of Left Vertebral Artery, Percutaneous Approach |
| 037Q4ZZ | Dilation of Left Vertebral Artery, Percutaneous Endoscopic Approach |
| Intracerebral Haemorrhage | |
| I61.0 | Nontraumatic intracerebral hemorrhage in hemisphere, subcortical |
| I61.1 | Nontraumatic intracerebral hemorrhage in hemisphere, cortical |
| I61.2 | Nontraumatic intracerebral hemorrhage in hemisphere, unspecified |
| I61.3 | Nontraumatic intracerebral hemorrhage in brain stem |
| I61.4 | Nontraumatic intracerebral hemorrhage in cerebellum |
| I61.5 | Nontraumatic intracerebral hemorrhage, intraventricular |
| I616 | Nontraumatic intracerebral hemorrhage, multiple localized |
| I618 | Other nontraumatic intracerebral hemorrhage |
| I619 | Nontraumatic intracerebral hemorrhage, unspecified |
| Subarachnoid haemorrhage | |
| 160.1 | Nontraumatic subarachnoid hemorrhage from middle cerebral |
| 160.2 | Nontraumatic subarachnoid hemorrhage from anterior communicating artery |
| 160.3 | Nontraumatic subarachnoid hemorrhage from posterior communicating artery |
| 160.4 | Nontraumatic subarachnoid hemorrhage from basilar artery |
| 160.5 | Nontraumatic subarachnoid hemorrhage from vertebral artery |
| 160.6 | Nontraumatic subarachnoid hemorrhage from other intracranial arteries |
| 160.8 | Other nontraumatic subarachnoid hemorrhage |
| 160.9 | Nontraumatic subarachnoid hemorrhage, unspecified |

ICD-10-CM: International Classification of Diseases-10th Revision-Clinical Modification, CPT: Current Procedural Terminology.

**Supplementary Table 2**. Baseline characteristics of older patients (>65 years) with stroke, comparing those with reduced versus normal albumin levels before and after propensity score matching.

|  | Before propensity score matching | | | After propensity score matching | | |
| --- | --- | --- | --- | --- | --- | --- |
|  | Reduced albumin levels  N = 1,659 | Normal albumin levels  N = 3,839 | ASD | Reduced albumin level  N = 1,593 | Normal albumin level  N = 1,593 | ASD |
| Age, y (± SD) | 76.4 ± 8.6 | 75.0 ± 8.6 | 0.161 | 76.2 ± 8.6 | 76.4 ± 8.6 | 0.028 |
| Female, n (%) | 874 (52.7) | 1,851 (48.2) | 0.089 | 833 (52.3) | 829 (52.0) | 0.005 |
| White, n (%) | 1,089 (65.6) | 2,219 (57.8) | 0.162 | 1,037 (65.1) | 1,055 (66.2) | 0.024 |
| Black or African American, n (%) | 208 (12.5) | 406 (10.6) | 0.061 | 196 (12.3) | 197 (12.4) | 0.002 |
| Asian, n (%) | 82 (4.9) | 335 (8.7) | 0.150 | 82 (5.1) | 74 (4.6) | 0.023 |
| Hypertension, n (%) | 530 (31.9) | 869 (22.6) | 0.210 | 477 (29.9) | 443 (27.8) | 0.047 |
| Ischaemic heart disease, n (%) | 215 (13.0) | 286 (7.4) | 0.183 | 182 (11.4) | 182 (11.4) | <0.001 |
| Atrial fibrillation, n (%) | 328 (19.8) | 432 (11.3) | 0.237 | 278 (17.5) | 252 (15.8) | 0.044 |
| Heart failure, n (%) | 173 (10.4) | 190 (4.9) | 0.207 | 136 (8.5) | 140 (8.8) | 0.009 |
| Pulmonary heart disease, n (%) | 54 (3.3) | 74 (1.9) | 0.084 | 45 (2.8) | 45 (2.8) | <0.001 |
| Lipoprotein disorder, n (%) | 342 (20.6) | 604 (15.7) | 0.127 | 311 (19.5) | 281 (17.6) | 0.048 |
| Diabetes mellitus, n (%) | 184 (11.1) | 271 (7.1) | 0.141 | 162 (10.2) | 148 (9.3) | 0.030 |
| Obesity, n (%) | 90 (5.4) | 174 (4.5) | 0.041 | 83 (5.2) | 75 (4.7) | 0.023 |
| Chronic kidney disease, n (%) | 107 (6.4) | 119 (3.1) | 0.158 | 86 (5.4) | 84 (5.3) | 0.006 |
| Cerebral infarction, n (%) | 557 (33.6) | 785 (20.4) | 0.299 | 492 (30.9) | 453 (28.4) | 0.054 |
| Peripheral vascular disease, n (%) | 38 (2.3) | 59 (1.5) | 0.055 | 33 (2.1) | 35 (2.2) | 0.009 |
| Symptoms and signs associated with systemic inflammation and infection, n (%) | 23 (1.4) | 13 (0.3) | 0.113 | 12 (0.8) | 13 (0.8) | 0.007 |
| Systemic connective tissue disorder (%) | 10 (0.6) | 19 (0.5) | 0.015 | 10 (0.6) | 10 (0.6) | <0.001 |
| Malnutrition, n (%) | 40 (2.4) | 23 (0.6) | 0.149 | 25 (1.6) | 21 (1.3) | 0.021 |
| Nephrotic syndrome, n (%) | 0 | 10 (0.3) | 0.072 | 0 | 0 | -- |
| Cirrhosis of liver, n (%) | 10 (0.6) | 10 (0.3) | 0.052 | 10 (0.6) | 10 (0.6) | <0.001 |
| Ulcerative colitis, n (%) | 10 (0.6) | 10 (0.3) | 0.052 | 10 (0.6) | 10 (0.6) | <0.001 |
| Crohn’s disease, n (%) | 10 (0.6) | 10 (0.3) | 0.052 | 10 (0.6) | 10 (0.6) | <0.001 |
| Burns and corrosions of external body surface, n (%) | 10 (0.6) | 10 (0.3) | 0.052 | 10 (0.6) | 10 (0.6) | <0.001 |
| NIHSS, n (%) | 269 (16.2) | 397 (10.3) | 0.174 | 240 (15.1) | 207 (13.0) | 0.060 |
| Echocardiography Procedures, n (%) | 112 (6.8) | 148 (3.9) | 0.129 | 100 (6.3) | 86 (5.4) | 0.037 |
| Cardiac Catheterization Procedures, n (%) | 11 (0.7) | 20 (0.5) | 0.019 | 10 (0.6) | 11 (0.7) | 0.008 |
| Electrocardiogram, routine ECG with at least 12 leads, n (%) | 335 (20.2) | 531 (13.8) | 0.170 | 303 (19.0) | 276 (17.3) | 0.044 |
| Antilipemic agents, n (%) | 308 (18.6) | 523 (13.8) | 0.135 | 275 (17.3) | 251 (15.8) | 0.041 |
| Beta blockers/related, n (%) | 429 (25.9) | 612 (15.9) | 0.246 | 373 (23.4) | 344 (21.6) | 0.044 |
| Antiarrhythmics, n (%) | 358 (21.6) | 500 (13.0) | 0.228 | 310 (19.5) | 296 (18.6) | 0.022 |
| Diuretics, n (%) | 141 (8.5) | 283 (7.4) | 0.042 | 130 (8.2) | 115 (7.2) | 0.035 |
| Calcium channel blockers, n (%) | 309 (18.6) | 483 (12.6) | 0.167 | 277 (17.4) | 247 (15.5) | 0.051 |
| Ace inhibitors, n (%) | 122 (7.4) | 219 (5.7) | 0.067 | 110 (6.9) | 98 (6.2) | 0.030 |
| Angiotensin II inhibitor, n (%) | 98 (5.9) | 176 (4.6) | 0.059 | 89 (5.6) | 79 (5.0) | 0.028 |
| Antianginals, n (%) | 86 (5.2) | 138 (3.6) | 0.078 | 75 (4.7) | 71 (4.5) | 0.012 |
| Anticoagulants, n (%) | 370 (22.3) | 454 (11.8) | 0.281 | 315 (19.8) | 301 (18.9) | 0.022 |
| Platelet aggregation inhibitors, n (%) | 232 (14.0) | 392 (10.2) | 0.116 | 207 (13.0) | 188 (11.8) | 0.036 |
| Alteplase, n (%) | 70 (4.2) | 106 (2.8) | 0.080 | 63 (4.0) | 63 (4.0) | <0.001 |
| Tenecteplase, n (%) | 27 (1.6) | 52 (1.4) | 0.023 | 25 (1.6) | 16 (1.0) | 0.050 |

ASD, absolute standardized mean difference; and HR, hazard ratio, NIHSS, National Institutes of Health Stroke Scale

**Supplementary Table 3**. Baseline characteristics of female patients with stroke, comparing those with reduced versus normal albumin levels before and after propensity score matching.

|  | Before propensity score matching | | | After propensity score matching | | |
| --- | --- | --- | --- | --- | --- | --- |
|  | Reduced albumin levels  N = 1,269 | Normal albumin levels  N = 2,744 | ASD | Reduced albumin level  N = 1,197 | Normal albumin level  N = 1,197 | ASD |
| Age, y (± SD) | 71.0 ± 14.9 | 69.9 ± 15.0 | 0.076 | 70.7 ± 14.9 | 70.7 ± 14.7 | <0.001 |
| Female, n (%) | 1,269 (100) | 2,744 (100) | -- | 1,197 (100) | 1,197 (100) | -- |
| White, n (%) | 813 (64.1) | 1,578 (57.5) | 0.135 | 757 (63.2) | 768 (64.2) | 0.019 |
| Black or African American, n (%) | 203 (16.0) | 393 (14.3) | 0.047 | 193 (16.1) | 207 (17.3) | 0.031 |
| Asian, n (%) | 64 (5.0) | 206 (7.5) | 0.102 | 62 (5.2) | 62 (5.2) | <0.001 |
| Hypertension, n (%) | 374 (29.5) | 574 (20.9) | 0.198 | 324 (27.1) | 299 (25.0) | 0.048 |
| Ischaemic heart disease, n (%) | 126 (9.9) | 147 (5.4) | 0.173 | 93 (7.8) | 88 (7.4) | 0.016 |
| Atrial fibrillation, n (%) | 229 (18.0) | 273 (9.9) | 0.235 | 187 (15.6) | 172 (14.4) | 0.035 |
| Heart failure, n (%) | 122 (9.6) | 125 (4.6) | 0.198 | 85 (7.1) | 85 (7.1) | <0.001 |
| Pulmonary heart disease, n (%) | 60 (4.7) | 57 (2.1) | 0.147 | 40 (3.3) | 42 (3.5) | 0.009 |
| Lipoprotein disorder, n (%) | 252 (19.9) | 386 (14.1) | 0.155 | 216 (18.0) | 210 (17.5) | 0.013 |
| Diabetes mellitus, n (%) | 140 (11.0) | 176 (6.4) | 0.164 | 109 (9.1) | 106 (8.9) | 0.009 |
| Obesity, n (%) | 95 (7.5) | 139 (5.1) | 0.100 | 76 (6.3) | 77 (6.4) | 0.003 |
| Chronic kidney disease, n (%) | 64 (5.0) | 75 (2.7) | 0.120 | 48 (4.0) | 44 (3.7) | 0.017 |
| Cerebral infarction, n (%) | 437 (34.4) | 535 (19.5) | 0.342 | 367 (30.7) | 343 (28.7) | 0.044 |
| Peripheral vascular disease, n (%) | 22 (1.7) | 39 (1.4) | 0.025 | 21 (1.8) | 17 (1.4) | 0.027 |
| Symptoms and signs associated with systemic inflammation and infection, n (%) | 14 (1.1) | 13 (0.5) | 0.071 | 11 (0.9) | 12 (1.0) | 0.009 |
| Systemic connective tissue disorder (%) | 10 (0.8) | 20 (0.7) | 0.007 | 10 (0.8) | 10 (0.8) | <0.001 |
| Malnutrition, n (%) | 34 (2.7) | 12 (0.4) | 0.182 | 10 (0.8) | 10 (0.8) | <0.001 |
| Nephrotic syndrome, n (%) | 0 | 0 | -- | 0 | 0 | -- |
| Cirrhosis of liver, n (%) | 10 (0.8) | 10 (0.4) | 0.056 | 10 (0.8) | 10 (0.8) | <0.001 |
| Ulcerative colitis, n (%) | 10 (0.8) | 10 (0.4) | 0.056 | 0 | 0 | -- |
| Crohn’s disease, n (%) | 10 (0.8) | 10 (0.4) | 0.056 | 10 (0.8) | 10 (0.8) | <0.001 |
| Burns and corrosions of external body surface, n (%) | 10 (0.8) | 10 (0.4) | 0.056 | 10 (0.8) | 10 (0.8) | <0.001 |
| NIHSS, n (%) | 210 (16.5) | 252 (9.2) | 0.221 | 173 (14.5) | 171 (14.3) | 0.005 |
| Echocardiography Procedures, n (%) | 84 (6.6) | 118 (4.3) | 0.102 | 77 (6.4) | 61 (5.1) | 0.057 |
| Cardiac Catheterization Procedures, n (%) | 10 (0.8) | 10 (0.4) | 0.056 | 10 (0.8) | 10 (0.8) | <0.001 |
| Electrocardiogram, routine ECG with at least 12 leads, n (%) | 270 (21.3) | 372 (13.6) | 0.205 | 238 (19.9) | 212 (17.7) | 0.056 |
| Antilipemic agents, n (%) | 240 (18.9) | 336 (12.2) | 0.185 | 203 (17.0) | 191 (16.0) | 0.027 |
| Beta blockers/related, n (%) | 325 (25.6) | 424 (15.5) | 0.254 | 273 (22.8) | 243 (20.3) | 0.061 |
| Antiarrhythmics, n (%) | 272 (21.4) | 327 (11.9) | 0.257 | 226 (18.9) | 208 (17.4) | 0.039 |
| Diuretics, n (%) | 118 (9.3) | 196 (7.1) | 0.079 | 101 (8.4) | 79 (6.6) | 0.070 |
| Calcium channel blockers, n (%) | 238 (18.8) | 327 (11.9) | 0.191 | 204 (17.0) | 197 (16.5) | 0.016 |
| Ace inhibitors, n (%) | 92 (7.2) | 135 (4.9) | 0.098 | 80 (6.7) | 68 (5.7) | 0.042 |
| Angiotensin II inhibitor, n (%) | 76 (6.0) | 120 (4.4) | 0.073 | 62 (5.2) | 69 (5.8) | 0.026 |
| Antianginals, n (%) | 66 (5.2) | 82 (3.0) | 0.112 | 54 (4.5) | 46 (3.8) | 0.033 |
| Anticoagulants, n (%) | 276 (21.7) | 314 (11.4) | 0.280 | 225 (18.8) | 214 (17.9) | 0.024 |
| Platelet aggregation inhibitors, n (%) | 166 (13.1) | 242 (8.8) | 0.137 | 146 (12.2) | 117 (9.8) | 0.078 |
| Alteplase, n (%) | 62 (4.9) | 80 (2.7) | 0.102 | 57 (4.8) | 52 (4.3) | 0.020 |
| Tenecteplase, n (%) | 22 (1.7) | 31 (1.1) | 0.051 | 19 (1.6) | 18 (1.5) | 0.007 |

ASD, absolute standardized mean difference; and HR, hazard ratio, NIHSS, National Institutes of Health Stroke Scale

**Supplementary Table 4**. Baseline characteristics of patients with mild to moderate strokes (NIHSS 0-15), comparing those with reduced versus normal albumin levels before and after propensity score matching.

|  | Before propensity score matching | | | After propensity score matching | | |
| --- | --- | --- | --- | --- | --- | --- |
|  | Reduced albumin levels  N = 662 | Normal albumin levels  N = 2,152 | ASD | Reduced albumin level  N = 642 | Normal albumin level  N = 642 | ASD |
| Age, y (± SD) | 67.7 ± 14.3 | 65.8 ± 14.3 | 0.133 | 67.4 ± 14.4 | 66.9 ± 14.3 | 0.038 |
| Female, n (%) | 303 (45.8) | 928 (43.1) | 0.053 | 295 (46.0) | 290 (45.2) | 0.016 |
| White, n (%) | 428 (64.7) | 1,348 (62.6) | 0.042 | 417 (65.0) | 416 (64.8) | 0.003 |
| Black or African American, n (%) | 95 (14.4) | 225 (10.5) | 0.118 | 87 (13.6) | 100 (15.6) | 0.057 |
| Asian, n (%) | 25 (3.8) | 84 (3.9) | 0.007 | 24 (3.7) | 24 (3.7) | <0.001 |
| Hypertension, n (%) | 215 (32.5) | 494 (23.0) | 0.214 | 199 (31.0) | 173 (26.9) | 0.089 |
| Ischaemic heart disease, n (%) | 92 (13.9) | 167 (7.8) | 0.198 | 77 (12.0) | 71 (11.1) | 0.029 |
| Atrial fibrillation, n (%) | 105 (15.9) | 200 (9.3) | 0.199 | 92 (14.3) | 75 (11.7) | 0.079 |
| Heart failure, n (%) | 73 (11.0) | 113 (5.3) | 0.212 | 58 (9.0) | 53 (8.3) | 0.028 |
| Pulmonary heart disease, n (%) | 30 (4.5) | 56 (2.6) | 0.104 | 24 (3.7) | 25 (3.9) | 0.008 |
| Lipoprotein disorder, n (%) | 147 (22.2) | 365 (17.0) | 0.132 | 133 (20.7) | 116 (18.1) | 0.067 |
| Diabetes mellitus, n (%) | 69 (10.4) | 151 (7.0) | 0.121 | 61 (9.5) | 54 (8.4) | 0.038 |
| Obesity, n (%) | 47 (7.1) | 134 (6.2) | 0.035 | 44 (6.9) | 46 (7.2) | 0.012 |
| Chronic kidney disease, n (%) | 38 (5.7) | 54 (2.5) | 0.163 | 28 (4.4) | 27 (4.2) | 0.008 |
| Cerebral infarction, n (%) | 211 (31.9) | 457 (21.2) | 0.243 | 192 (29.9) | 171 (26.6) | 0.073 |
| Peripheral vascular disease, n (%) | 15 (2.3) | 28 (1.3) | 0.073 | 13 (2.0) | 10 (1.6) | 0.035 |
| Symptoms and signs associated with systemic inflammation and infection, n (%) | 11 (1.7) | 10 (0.5) | 0.117 | 10 (1.6) | 10 (1.6) | <0.001 |
| Systemic connective tissue disorder (%) | 10 (1.5) | 10 (0.5) | 0.106 | 10 (1.6) | 10 (1.6) | <0.001 |
| Malnutrition, n (%) | 15 (2.3) | 14 (0.7) | 0.135 | 10 (1.6) | 10 (1.6) | <0.001 |
| Nephrotic syndrome, n (%) | 0 | 10 (0.5) | 0.097 | 0 | 0 | -- |
| Cirrhosis of liver, n (%) | 10 (1.5) | 10 (0.5) | 0.106 | 10 (1.6) | 10 (1.6) | <0.001 |
| Ulcerative colitis, n (%) | 10 (1.5) | 0 | 0.175 | 0 | 0 | -- |
| Crohn’s disease, n (%) | 10 (1.5) | 10 (0.5) | 0.106 | 10 (1.6) | 10 (1.6) | <0.001 |
| Burns and corrosions of external body surface, n (%) | 10 (1.5) | 10 (0.5) | 0.106 | 10 (1.6) | 10 (1.6) | <0.001 |
| NIHSS, n (%) | 190 (28.7) | 369 (17.1) | 0.278 | 171 (26.6) | 157 (24.5) | 0.050 |
| Echocardiography Procedures, n (%) | 32 (4.8) | 89 (4.1) | 0.034 | 31 (4.8) | 27 (4.2) | 0.030 |
| Cardiac Catheterization Procedures, n (%) | 10 (1.5) | 10 (0.5) | 0.106 | 10 (1.6) | 10 (1.6) | <0.001 |
| Electrocardiogram, routine ECG with at least 12 leads, n (%) | 152 (23.0) | 349 (16.2) | 0.171 | 140 (21.8) | 127 (19.8) | 0.050 |
| Antilipemic agents, n (%) | 113 (17.1) | 249 (11.6) | 0.157 | 102 (15.9) | 92 (14.3) | 0.044 |
| Beta blockers/related, n (%) | 149 (22.5) | 331 (15.4) | 0.183 | 138 (21.5) | 129 (20.1) | 0.035 |
| Antiarrhythmics, n (%) | 135 (20.4) | 308 (14.3) | 0.161 | 127 (19.8) | 117 (18.2) | 0.040 |
| Diuretics, n (%) | 43 (6.5) | 116 (5.4) | 0.047 | 39 (6.1) | 37 (5.8) | 0.013 |
| Calcium channel blockers, n (%) | 110 (16.6) | 234 (10.9) | 0.167 | 106 (16.5) | 96 (15.0) | 0.043 |
| Ace inhibitors, n (%) | 42 (6.3) | 99 (4.6) | 0.077 | 42 (6.5) | 39 (6.1) | 0.019 |
| Angiotensin II inhibitor, n (%) | 23 (3.5) | 76 (3.5) | 0.003 | 22 (3.4) | 15 (2.3) | 0.065 |
| Antianginals, n (%) | 33 (5.0) | 64 (3.0) | 0.103 | 27 (4.2) | 21 (3.3) | 0.049 |
| Anticoagulants, n (%) | 124 (18.7) | 239 (11.1) | 0.215 | 109 (17.0) | 106 (16.5) | 0.013 |
| Platelet aggregation inhibitors, n (%) | 77 (11.6) | 194 (9.0) | 0.086 | 70 (10.9) | 58 (9.0) | 0.062 |
| Alteplase, n (%) | 36 (5.4) | 63 (2.9) | 0.126 | 33 (5.1) | 32 (5.0) | 0.007 |
| Tenecteplase, n (%) | 21 (3.2) | 42 (2.0) | 0.077 | 19 (3.0) | 19 (3.0) | <0.001 |

ASD, absolute standardized mean difference; and HR, hazard ratio, NIHSS, National Institutes of Health Stroke Scale

**Supplementary Table 5**. Baseline characteristics of patients with severe strokes (NIHSS 16-42), comparing those with reduced versus normal albumin levels before and after propensity score matching.

|  | Before propensity score matching | | | After propensity score matching | | |
| --- | --- | --- | --- | --- | --- | --- |
|  | Reduced albumin levels  N = 713 | Normal albumin levels  N = 1,733 | ASD | Reduced albumin level  N = 664 | Normal albumin level  N = 664 | ASD |
| Age, y (± SD) | 71.2 ± 14.2 | 68.9 ± 14.3 | 0.162 | ± | ± | 0.025 |
| Female, n (%) | 378 (53.0) | 778 (44.9) | 0.163 | 343 (51.7) | 337 (50.8) | 0.018 |
| White, n (%) | 471 (66.1) | 997 (57.5) | 0.176 | 430 (64.8) | 434 (65.4) | 0.013 |
| Black or African American, n (%) | 80 (11.2) | 176 (10.2) | 0.034 | 77 (11.6) | 71 (10.7) | 0.029 |
| Asian, n (%) | 26 (3.6) | 85 (4.9) | 0.062 | 26 (3.9) | 23 (3.5) | 0.024 |
| Hypertension, n (%) | 225 (31.6) | 402 (23.2) | 0.188 | 187 (28.2) | 176 (26.5) | 0.037 |
| Ischaemic heart disease, n (%) | 105 (14.7) | 134 (7.7) | 0.223 | 81 (12.2) | 77 (11.6) | 0.019 |
| Atrial fibrillation, n (%) | 126 (17.7) | 189 (10.9) | 0.194 | 98 (14.8) | 91 (13.7) | 0.030 |
| Heart failure, n (%) | 91 (12.8) | 106 (6.1) | 0.229 | 70 (10.5) | 69 (10.4) | 0.005 |
| Pulmonary heart disease, n (%) | 36 (5.0) | 38 (2.2) | 0.153 | 25 (3.8) | 28 (4.2) | 0.023 |
| Lipoprotein disorder, n (%) | 153 (21.5) | 284 (16.4) | 0.130 | 130 (19.6) | 118 (17.8) | 0.046 |
| Diabetes mellitus, n (%) | 64 (9.0) | 120 (6.9) | 0.076 | 54 (8.1) | 51 (7.7) | 0.017 |
| Obesity, n (%) | 49 (6.9) | 82 (4.7) | 0.092 | 40 (6.0) | 38 (5.7) | 0.013 |
| Chronic kidney disease, n (%) | 49 (6.9) | 57 (3.3) | 0.164 | 35 (5.3) | 34 (5.1) | 0.007 |
| Cerebral infarction, n (%) | 227 (31.8) | 374 (21.6) | 0.233 | 183 (27.6) | 170 (25.6) | 0.044 |
| Peripheral vascular disease, n (%) | 11 (1.5) | 27 (1.6) | 0.001 | 10 (1.5) | 10 (1.5) | <0.001 |
| Symptoms and signs associated with systemic inflammation and infection, n (%) | 12 (1.7) | 16 (0.9) | 0.067 | 10 (1.5) | 10 (1.5) | <0.001 |
| Systemic connective tissue disorder (%) | 10 (1.4) | 11 (0.6) | 0.077 | 10 (1.5) | 10 (1.5) | <0.001 |
| Malnutrition, n (%) | 27 (3.8) | 10 (0.6) | 0.221 | 10 (1.5) | 10 (1.5) | <0.001 |
| Nephrotic syndrome, n (%) | 0 | 0 | -- | 0 | 0 | -- |
| Cirrhosis of liver, n (%) | 10 (1.4) | 10 (0.6) | 0.083 | 10 (1.5) | 10 (1.5) | <0.001 |
| Ulcerative colitis, n (%) | 10 (1.4) | 10 (0.6) | 0.083 | 10 (1.5) | 10 (1.5) | <0.001 |
| Crohn’s disease, n (%) | 10 (1.4) | 10 (0.6) | 0.083 | 10 (1.5) | 10 (1.5) | <0.001 |
| Burns and corrosions of external body surface, n (%) | 10 (1.4) | 10 (0.6) | 0.083 | 10 (1.5) | 10 (1.5) | <0.001 |
| NIHSS, n (%) | 204 (28.6) | 314 (18.1) | 0.250 | 163 (24.5) | 154 (23.2) | 0.032 |
| Echocardiography Procedures, n (%) | 49 (6.9) | 62 (3.6) | 0.148 | 38 (5.7) | 36 (5.4) | 0.013 |
| Cardiac Catheterization Procedures, n (%) | 10 (1.4) | 10 (0.6) | 0.083 | 10 (1.5) | 10 (1.5) | <0.001 |
| Electrocardiogram, routine ECG with at least 12 leads, n (%) | 150 (21.0) | 240 (13.8) | 0.190 | 121 (18.2) | 111 (16.7) | 0.040 |
| Antilipemic agents, n (%) | 131 (18.4) | 224 (12.9) | 0.150 | 110 (16.6) | 110 (16.6) | <0.001 |
| Beta blockers/related, n (%) | 175 (24.5) | 270 (15.6) | 0.225 | 143 (21.5) | 144 (21.7) | 0.004 |
| Antiarrhythmics, n (%) | 128 (18.0) | 207 (11.9) | 0.169 | 105 (15.8) | 90 (13.6) | 0.064 |
| Diuretics, n (%) | 63 (8.8) | 105 (6.1) | 0.106 | 53 (8.0) | 57 (8.6) | 0.022 |
| Calcium channel blockers, n (%) | 114 (16.0) | 199 (11.5) | 0.131 | 94 (14.2) | 95 (14.3) | 0.004 |
| Ace inhibitors, n (%) | 56 (7.9) | 84 (4.8) | 0.124 | 46 (6.9) | 45 (6.8) | 0.006 |
| Angiotensin II inhibitor, n (%) | 34 (4.8) | 75 (4.3) | 0.021 | 31 (4.7) | 30 (4.5) | 0.007 |
| Antianginals, n (%) | 42 (5.9) | 66 (3.8) | 0.097 | 36 (5.4) | 31 (4.7) | 0.034 |
| Anticoagulants, n (%) | 142 (19.9) | 185 (10.7) | 0.259 | 112 (16.9) | 107 (16.1) | 0.020 |
| Platelet aggregation inhibitors, n (%) | 82 (11.5) | 155 (8.9) | 0.084 | 75 (11.3) | 70 (10.5) | 0.024 |
| Alteplase, n (%) | 28 (3.9) | 56 (3.2) | 0.037 | 24 (3.6) | 24 (3.6) | <0.001 |
| Tenecteplase, n (%) | 21 (2.9) | 33 (1.9) | 0.068 | 19 (2.9) | 19 (2.9) | <0.001 |

ASD, absolute standardized mean difference; and HR, hazard ratio, NIHSS, National Institutes of Health Stroke Scale

**Supplementary Table 6**. Baseline characteristics of patients without protein-calorie malnutrition, comparing those with reduced versus normal albumin levels before and after propensity score matching.

|  | Before propensity score matching | | | After propensity score matching | | |
| --- | --- | --- | --- | --- | --- | --- |
|  | Reduced albumin levels  N = 2,228 | Normal albumin levels  N = 5,845 | ASD | Reduced albumin level  N = 2,158 | Normal albumin level  N = 2,158 | ASD |
| Age, y (± SD) | 70.0 ± 14.2 | 67.1 ± 14.5 | 0.206 | 69.7 ± 14.2 | 70.1 ± 14.0 | 0.027 |
| Female, n (%) | 1,152 (51.7) | 2,575 (44.1) | 0.154 | 1,102 (51.1) | 1,127 (52.2) | 0.023 |
| White, n (%) | 1,381 (62.0) | 3,306 (56.6) | 0.111 | 1,330 (61.6) | 1,349 (62.5) | 0.018 |
| Black or African American, n (%) | 342 (15.4) | 836 (14.3) | 0.029 | 330 (15.3) | 335 (15.5) | 0.006 |
| Asian, n (%) | 124 (5.6) | 477 (8.2) | 0.103 | 123 (5.7) | 119 (5.5) | 0.008 |
| Hypertension, n (%) | 621 (27.9) | 1,198 (20.05) | 0.173 | 570 (26.4) | 527 (24.4) | 0.046 |
| Ischaemic heart disease, n (%) | 248 (11.1) | 377 (6.4) | 0.166 | 212 (9.8) | 200 (9.3) | 0.019 |
| Atrial fibrillation, n (%) | 351 (15.8) | 518 (8.9) | 0.211 | 311 (14.4) | 287 (13.3) | 0.032 |
| Heart failure, n (%) | 213 (9.6) | 274 (4.7) | 0.190 | 179 (8.3) | 176 (8.2) | 0.005 |
| Pulmonary heart disease, n (%) | 78 (3.5) | 108 (1.8) | 0.103 | 67 (3.1) | 58 (2.7) | 0.025 |
| Lipoprotein disorder, n (%) | 418 (18.8) | 812 (13.9) | 0.132 | 387 (17.9) | 337 (15.6) | 0.062 |
| Diabetes mellitus, n (%) | 233 (10.5) | 395 (6.8) | 0.132 | 241 (9.9) | 185 (8.6) | 0.046 |
| Obesity, n (%) | 132 (5.9) | 286 (4.9) | 0.046 | 124 (5.7) | 99 (4.6) | 0.052 |
| Chronic kidney disease, n (%) | 119 (5.3) | 144 (2.5) | 0.149 | 97 (4.5) | 98 (4.5) | 0.002 |
| Cerebral infarction, n (%) | 720 (32.3) | 1,182 (20.2) | 0.277 | 653 (30.3) | 594 (27.5) | 0.060 |
| Peripheral vascular disease, n (%) | 51 (2.3) | 74 (1.3) | 0.077 | 45 (2.1) | 40 (1.9) | 0.017 |
| Symptoms and signs associated with systemic inflammation and infection, n (%) | 26 (1.2) | 24 (0.4) | 0.086 | 18 (0.8) | 19 (0.9) | 0.005 |
| Systemic connective tissue disorder (%) | 10 (0.4) | 26 (0.4) | 0.001 | 10 (0.5) | 10 (0.5) | <0.001 |
| Malnutrition, n (%) | 37 (1.7) | 18 (0.3) | 0.137 | 18 (0.8) | 17 (0.8) | 0.005 |
| Nephrotic syndrome, n (%) | 0 | 10 (0.2) | 0.059 | 0 | 0 | -- |
| Cirrhosis of liver, n (%) | 10 (0.4) | 10 (0.2) | 0.050 | 10 (0.5) | 10 (0.5) | <0.001 |
| Ulcerative colitis, n (%) | 10 (0.4) | 10 (0.2) | 0.050 | 10 (0.5) | 10 (0.5) | <0.001 |
| Crohn’s disease, n (%) | 10 (0.4) | 10 (0.2) | 0.050 | 10 (0.5) | 10 (0.5) | <0.001 |
| Burns and corrosions of external body surface, n (%) | 10 (0.4) | 10 (0.2) | 0.050 | 10 (0.5) | 10 (0.5) | <0.001 |
| NIHSS, n (%) | 366 (16.4) | 621 (10.6) | 0.170 | 322 (14.9) | 289 (13.4) | 0.044 |
| Echocardiography Procedures, n (%) | 131 (5.9) | 199 (3.4) | 0.118 | 118 (5.5) | 107 (5.0) | 0.023 |
| Cardiac Catheterization Procedures, n (%) | 18 (0.8) | 24 (0.4) | 0.051 | 13 (0.6) | 14 (0.6) | 0.006 |
| Electrocardiogram, routine ECG with at least 12 leads, n (%) | 431 (19.3) | 751 (12.8) | 0.177 | 394 (18.3) | 353 (16.4) | 0.050 |
| Antilipemic agents, n (%) | 361 (16.2) | 677 (11.6) | 0.134 | 332 (15.4) | 292 (13.5) | 0.053 |
| Beta blockers/related, n (%) | 506 (22.7) | 819 (14.0) | 0.226 | 456 (21.1) | 418 (19.4) | 0.044 |
| Antiarrhythmics, n (%) | 451 (20.2) | 709 (12.1) | 0.222 | 401 (18.6) | 363 (16.8) | 0.046 |
| Diuretics, n (%) | 172 (7.7) | 357 (6.1) | 0.064 | 161 (7.5) | 150 (7.0) | 0.020 |
| Calcium channel blockers, n (%) | 347 (15.6) | 632 (10.8) | 0.141 | 326 (15.1) | 292 (13.5) | 0.045 |
| Ace inhibitors, n (%) | 140 (6.3) | 277 (4.7) | 0.068 | 132 (6.1) | 107 (5.0) | 0.051 |
| Angiotensin II inhibitor, n (%) | 110 (4.9) | 224 (3.8) | 0.054 | 105 (4.9) | 97 (4.5) | 0.018 |
| Antianginals, n (%) | 108 (4.8) | 188 (3.2) | 0.083 | 94 (4.4) | 81 (3.8) | 0.031 |
| Anticoagulants, n (%) | 439 (19.7) | 628 (10.7) | 0.251 | 385 (17.8) | 369 (17.1) | 0.020 |
| Platelet aggregation inhibitors, n (%) | 281 (12.6) | 543 (9.3) | 0.107 | 260 (12.0) | 233 (10.8) | 0.039 |
| Alteplase, n (%) | 90 (4.0) | 163 (2.8) | 0.069 | 84 (3.9) | 70 (3.2) | 0.035 |
| Tenecteplase, n (%) | 40 (1.8) | 71 (1.2) | 0.048 | 36 (1.7) | 33 (1.5) | 0.011 |

ASD, absolute standardized mean difference; and HR, hazard ratio, NIHSS, National Institutes of Health Stroke Scale

**Supplementary Table 7**. Baseline characteristics of patients with mild hypalbuminaemia versus patients with normal albumin levels before and after propensity score matching.

|  | Before propensity score matching | | | After propensity score matching | | |
| --- | --- | --- | --- | --- | --- | --- |
|  | Reduced albumin levels  N = 2,362 | Normal albumin levels  N = 5,609 | ASD | Reduced albumin level  N = 2,302 | Normal albumin level  N = 2,302 | ASD |
| Age, y (± SD) | 69.9 ± 14.1 | 67.2 ± 14.5 | 0.187 | 69.7 ± 14.1 | 69.5 ± 14.2 | 0.013 |
| Female, n (%) | 1,196 (50.6) | 2,491 (44.4) | 0.125 | 1,158 (50.3) | 1,169 (50.8) | 0.010 |
| White, n (%) | 1,473 (62.4) | 3,112 (55.5) | 0.140 | 1,426 (61.9) | 1,445 (62.8) | 0.017 |
| Black or African American, n (%) | 346 (14.6) | 809 (14.4) | 0.006 | 335 (14.6) | 354 (15.4) | 0.023 |
| Asian, n (%) | 141 (6.0) | 480 (8.6) | 0.100 | 141 (6.1) | 136 (5.9) | 0.009 |
| Hypertension, n (%) | 687 (29.1) | 1,183 (21.1) | 0.185 | 636 (27.6) | 606 (26.3) | 0.029 |
| Ischaemic heart disease, n (%) | 271 (11.5) | 368 (6.6) | 0.172 | 232 (10.1) | 233 (10.1) | 0.001 |
| Atrial fibrillation, n (%) | 387 (16.4) | 513 (9.1) | 0.218 | 343 (14.9) | 331 (14.4) | 0.015 |
| Heart failure, n (%) | 225 (9.5) | 273 (4.9) | 0.181 | 191 (8.3) | 191 (8.3) | <0.001 |
| Pulmonary heart disease, n (%) | 79 (3.3) | 102 (1.8) | 0.096 | 69 (3.0) | 72 (3.1) | 0.008 |
| Lipoprotein disorder, n (%) | 447 (18.9) | 798 (14.2) | 0.127 | 415 (18.0) | 380 (16.5) | 0.040 |
| Diabetes mellitus, n (%) | 239 (10.1) | 389 (6.9) | 0.114 | 217 (9.4) | 204 (8.9) | 0.020 |
| Obesity, n (%) | 143 (6.1) | 277 (4.9) | 0.049 | 139 (6.0) | 127 (5.5) | 0.022 |
| Chronic kidney disease, n (%) | 125 (5.3) | 142 (2.5) | 0.143 | 101 (4.4) | 96 (4.2) | 0.011 |
| Cerebral infarction, n (%) | 752 (31.8) | 1,162 (20.7) | 0.255 | 698 (30.3) | 666 (28.9) | 0.030 |
| Peripheral vascular disease, n (%) | 52 (2.2) | 76 (1.4) | 0.064 | 46 (2.0) | 45 (2.0) | 0.003 |
| Symptoms and signs associated with systemic inflammation and infection, n (%) | 29 (1.2) | 22 (0.4) | 0.093 | 17 (0.7) | 21 (0.9) | 0.019 |
| Systemic connective tissue disorder (%) | 10 (0.4) | 26 (0.5) | 0.006 | 10 (0.4) | 10 (0.4) | <0.001 |
| Malnutrition, n (%) | 43 (1.8) | 27 (0.5) | 0.126 | 25 (1.1) | 25 (1.1) | <0.001 |
| Nephrotic syndrome, n (%) | 0 | 10 (0.2) | 0.060 | 0 | 0 | -- |
| Cirrhosis of liver, n (%) | 10 (0.4) | 10 (0.2) | 0.045 | 10 (0.4) | 10 (0.4) | <0.001 |
| Ulcerative colitis, n (%) | 10 (0.4) | 10 (0.2) | 0.045 | 10 (0.4) | 10 (0.4) | <0.001 |
| Crohn’s disease, n (%) | 10 (0.4) | 10 (0.2) | 0.045 | 10 (0.4) | 10 (0.4) | <0.001 |
| Burns and corrosions of external body surface, n (%) | 10 (0.4) | 10 (0.2) | 0.045 | 10 (0.4) | 10 (0.4) | <0.001 |
| NIHSS, n (%) | 401 (17.0) | 617 (11.0) | 0.173 | 372 (16.2) | 353 (15.3) | 0.023 |
| Echocardiography Procedures, n (%) | 143 (6.1) | 194 (3.5) | 0.122 | 127 (5.5) | 124 (5.4) | 0.006 |
| Cardiac Catheterization Procedures, n (%) | 19 (0.8) | 23 (0.4) | 0.051 | 17 (0.7) | 18 (0.8) | 0.005 |
| Electrocardiogram, routine ECG with at least 12 leads, n (%) | 471 (19.9) | 741 (13.2) | 0.182 | 436 (18.9) | 431 (18.7) | 0.006 |
| Antilipemic agents, n (%) | 383 (16.2) | 648 (11.6) | 0.135 | 354 (15.4) | 341 (14.8) | 0.016 |
| Beta blockers/related, n (%) | 540 (22.9) | 807 (14.4) | 0.219 | 497 (21.6) | 475 (20.6) | 0.023 |
| Antiarrhythmics, n (%) | 465 (19.7) | 705 (12.6) | 0.194 | 426 (18.5) | 405 (17.6) | 0.024 |
| Diuretics, n (%) | 199 (8.4) | 342 (6.1) | 0.090 | 185 (8.0) | 181(7.9) | 0.006 |
| Calcium channel blockers, n (%) | 380 (16.1) | 627 (11.2) | 0.143 | 354 (15.4) | 343 (14.9) | 0.013 |
| Ace inhibitors, n (%) | 151 (6.4) | 264 (4.7) | 0.074 | 144 (6.3) | 128 (5.6) | 0.029 |
| Angiotensin II inhibitor, n (%) | 126 (5.3) | 206 (3.7) | 0.080 | 115 (5.0) | 111 (4.8) | 0.008 |
| Antianginals, n (%) | 121 (5.1) | 184 (3.3) | 0.092 | 101 (4.4) | 111 (4.8) | 0.021 |
| Anticoagulants, n (%) | 435 (18.4) | 607 (10.8) | 0.216 | 391 (17.0) | 380 (16.5) | 0.013 |
| Platelet aggregation inhibitors, n (%) | 301 (12.7) | 529 (9.4) | 0.106 | 278 (12.1) | 271 (11.8) | 0.009 |
| Alteplase, n (%) | 105 (4.4) | 162 (2.9) | 0.083 | 101 (4.4) | 93 (4.0) | 0.017 |
| Tenecteplase, n (%) | 44 (1.9) | 75 (1.3) | 0.042 | 41 (1.8) | 39 (1.7) | 0.007 |

ASD, absolute standardized mean difference; and HR, hazard ratio, NIHSS, National Institutes of Health Stroke Scale

**Supplementary Table 8**. Baseline characteristics of patients with severe hypalbuminaemia versus patients with normal albumin levels before and after propensity score matching.

|  | Before propensity score matching | | | After propensity score matching | | |
| --- | --- | --- | --- | --- | --- | --- |
|  | Reduced albumin levels  N = 413 | Normal albumin levels  N = 6,010 | ASD | Reduced albumin level  N = 402 | Normal albumin level  N = 402 | ASD |
| Age, y (± SD) | 68.2 ± 14.3 | 67.1 ± 14.5 | 0.073 | 68.1 ± 14.4 | 69.5 ± 14.3 | 0.100 |
| Female, n (%) | 215 (52.1) | 2,656 (44.2) | 0.158 | 207 (51.5) | 205 (51.0) | 0.010 |
| White, n (%) | 267 (64.6) | 3,407 (56.7) | 0.163 | 259 (64.4) | 258 (64.2) | 0.005 |
| Black or African American, n (%) | 65 (15.7) | 867 (14.4) | 0.037 | 62 (15.4) | 67 (16.7) | 0.034 |
| Asian, n (%) | 18 (4.4) | 485 (8.1) | 0.154 | 18 (4.5) | 20 (5.0) | 0.023 |
| Hypertension, n (%) | 112 (27.1) | 1,232 (20.5) | 0.156 | 102 (25.4) | 93 (23.1) | 0.052 |
| Ischaemic heart disease, n (%) | 50 (12.1) | 386 (6.4) | 0.197 | 44 (10.9) | 45 (11.2) | 0.008 |
| Atrial fibrillation, n (%) | 54 (13.1) | 532 (8.9) | 0.135 | 46 (11.4) | 50 (12.4) | 0.031 |
| Heart failure, n (%) | 41 (9.9) | 282 (4.7) | 0.202 | 36 (9.0) | 37 (9.2) | 0.009 |
| Pulmonary heart disease, n (%) | 26 (6.3) | 110 (1.8) | 0.228 | 19 (4.7) | 17 (4.2) | 0.024 |
| Lipoprotein disorder, n (%) | 74 (17.9) | 838 (13.9) | 0.109 | 69 (17.2) | 62 (15.4) | 0.047 |
| Diabetes mellitus, n (%) | 42 (10.2) | 406 (6.8) | 0.123 | 39 (9.7) | 32 (8.0) | 0.061 |
| Obesity, n (%) | 27 (6.5) | 293 (4.9) | 0.072 | 26 (6.5) | 22 (5.5) | 0.042 |
| Chronic kidney disease, n (%) | 31 (7.5) | 149 (2.5) | 0.232 | 25 (6.2) | 26 (6.5) | 0.010 |
| Cerebral infarction, n (%) | 124 (30.0) | 1,215 (20.2) | 0.228 | 113 (28.1) | 102 (25.4) | 0.062 |
| Peripheral vascular disease, n (%) | 10 (2.4) | 78 (1.3) | 0.083 | 10 (2.5) | 11 (2.7) | 0.016 |
| Symptoms and signs associated with systemic inflammation and infection, n (%) | 10 (2.4) | 27 (0.4) | 0.166 | 10 (2.5) | 10 (2.5) | <0.001 |
| Systemic connective tissue disorder (%) | 10 (2.4) | 28 (0.5) | 0.164 | 10 (2.5) | 0 | 0.226 |
| Malnutrition, n (%) | 20 (4.8) | 29 (0.5) | 0.273 | 13 (3.2) | 11 (2.7) | 0.029 |
| Nephrotic syndrome, n (%) | 0 | 10 (0.2) | 0.058 | 0 | 0 | -- |
| Cirrhosis of liver, n (%) | 10 (2.4) | 10 (0.2) | 0.201 | 10 (2.5) | 10 (2.5) | <0.001 |
| Ulcerative colitis, n (%) | 0 | 10 (0.2) | 0.058 | 0 | 0 | -- |
| Crohn’s disease, n (%) | 10 (2.4) | 10 (0.2) | 0.201 | 10 (2.5) | 10 (2.5) | <0.001 |
| Burns and corrosions of external body surface, n (%) | 10 (2.4) | 10 (0.2) | 0.201 | 10 (2.5) | 10 (2.5) | <0.001 |
| NIHSS, n (%) | 67 (16.2) | 643 (10.7) | 0.162 | 62 (15.4) | 53 (13.2) | 0.064 |
| Echocardiography Procedures, n (%) | 24 (5.8) | 204 (3.4) | 0.116 | 23 (5.7) | 15 (3.7) | 0.094 |
| Cardiac Catheterization Procedures, n (%) | 10 (2.4) | 24 (0.4) | 0.172 | 10 (2.5) | 0 | 0.226 |
| Electrocardiogram, routine ECG with at least 12 leads, n (%) | 71 (17.2) | 780 (13.0) | 0.118 | 68 (16.9) | 52 (12.9) | 0.112 |
| Antilipemic agents, n (%) | 63 (15.3) | 695 (11.6) | 0.108 | 59 (14.7) | 51 (12.7) | 0.058 |
| Beta blockers/related, n (%) | 88 (21.3) | 847 (14.1) | 0.190 | 82 (20.4) | 78 (19.4) | 0.025 |
| Antiarrhythmics, n (%) | 76 (18.4) | 731 (12.2) | 0.174 | 71 (17.7) | 51 (12.7) | 0.139 |
| Diuretics, n (%) | 25 (6.1) | 364 (6.1) | <0.001 | 23 (5.7) | 14 (3.5) | 0.107 |
| Calcium channel blockers, n (%) | 59 (14.3) | 649 (10.8) | 0.105 | 57 (14.2) | 44 (10.9) | 0.098 |
| Ace inhibitors, n (%) | 18 (4.4) | 285 (4.7) | 0.018 | 17 (4.2) | 18 (4.5) | 0.012 |
| Angiotensin II inhibitor, n (%) | 10 (2.4) | 228 (3.8) | 0.079 | 10 (2.5) | 10 (2.5) | <0.001 |
| Antianginals, n (%) | 15 (3.6) | 197 (3.3) | 0.019 | 15 (3.7) | 10 (2.5) | 0.072 |
| Anticoagulants, n (%) | 77 (18.6) | 642 (10.7) | 0.227 | 75 (18.7) | 62 (15.4) | 0.086 |
| Platelet aggregation inhibitors, n (%) | 45 (10.9) | 559 (9.3) | 0.053 | 42 (10.4) | 36 (9.0) | 0.050 |
| Alteplase, n (%) | 10 (2.4) | 167 (2.8) | 0.022 | 10 (2.5) | 11 (2.7) | 0.016 |
| Tenecteplase, n (%) | 10 (2.4) | 76 (1.3) | 0.086 | 10 (2.5) | 10 (2.5) | <0.001 |

ASD, absolute standardized mean difference; and HR, hazard ratio, NIHSS, National Institutes of Health Stroke Scale

**Supplementary Table 9**. Baseline characteristics of patients with acute ischaemic stroke who did not undergo endovascular thrombectomy before and after propensity score matching.

|  | Before propensity score matching | | | After propensity score matching | | |
| --- | --- | --- | --- | --- | --- | --- |
|  | Reduced albumin levels  N = 37,404 | Normal albumin levels  N = 122,849 | ASD | Reduced albumin level  N = 36,498 | Normal albumin level  N = 36,498 | ASD |
| Age, y (± SD) | 68 ± 15 | 66 ± 15 | 0.164 | 68 ±15 | 68 ± 15 |  |
| Female, n (%) | 17,211 (46.0) | 52,500 (42.7) | 0.066 | 16,776 (46.0) | 16,830 (46.1) |  |
| White, n (%) | 20,997 (56.1) | 68,736 (56.0) | 0.004 | 20,502 (56.2) | 20,510 (56.2) |  |
| Black or African American, n (%) | 7,232 (19.3) | 22,320 (18.2) | 0.030 | 7,014 (19.2) | 6,926 (19.0) |  |
| Asian, n (%) | 1,519 (4.1) | 7,924 (6.5) | 0.107 | 1,497 (4.1) | 1,464 (4.0) |  |
| Hypertension, n (%) | 11,104 (29.7) | 31,238 (25.4) | 0.095 | 10,389 (28.5) | 9,835 (26.9) |  |
| Ischaemic heart disease, n (%) | 4,628 (12.4) | 9,341 (7.6) | 0.160 | 4,414 (11.3) | 4,037 (11.1) |  |
| Atrial fibrillation, n (%) | 3,438 (9.2) | 5,946 (4.8) | 0.171 | 3,007 (8.2) | 3,017 (8.3) |  |
| Heart failure, n (%) | 2,976 (8.0) | 4,059 (3.3) | 0.203 | 2,498 (6.8) | 2,490 (6.8) |  |
| Pulmonary heart disease, n (%) | 1,156 (3.1) | 1,522 (1.2) | 0.127 | 938 (2.6) | 910 (2.5) |  |
| Lipoprotein disorder, n (%) | 6,919 (18.5) | 20,772 (16.9) | 0.042 | 6,507 (17.8) | 6,072 (16.6) |  |
| Diabetes mellitus, n (%) | 5,603 (15.0) | 13,093 (10.7) | 0.130 | 5,166 (14.2) | 4,967 (13.6) |  |
| Obesity, n (%) | 2,432 (6.5) | 6,463 (5.3) | 0.053 | 2,240 (6.1) | 2,076 (5.7) |  |
| Chronic kidney disease, n (%) | 2,895 (7.7) | 4,413 (3.6) | 0.180 | 2,487 (6.8) | 2,466 (6.8) |  |
| Cerebral infarction, n (%) | 9,660 (25.8) | 21,287 (17.3) | 0.208 | 8,846 (24.2) | 8,411 (23.0) |  |
| Peripheral vascular disease, n (%) | 978 (2.6) | 2,060 (1.7) | 0.065 | 880 (2.4) | 859 (2.4) |  |
| Symptoms and signs associated with systemic inflammation and infection, n (%) | 850 (2.3) | 407 (0.3) | 0.172 | 433 (1.2) | 383 (0.078) |  |
| Systemic connective tissue disorder (%) | 303 (0.8) | 647 (0.5) | 0.035 | 271 (0.7) | 241 (0.7) |  |
| Malnutrition, n (%) | 1,230 (3.3) | 707 (0.6) | 0.198 | 719 (2.0) | 664 (1.8) |  |
| Nephrotic syndrome, n (%) | 10 (0.0) | 10 (0.0) | 0.014 | 10 (0.0) | 10 (0.0) |  |
| Cirrhosis of liver, n (%) | 215 (0.6) | 214 (0.2) | 0.066 | 153 (0.4) | 155 (0.4) |  |
| Ulcerative colitis, n (%) | 40 (0.1) | 139 (0.1) | 0.002 | 39 (0.1) | 38 (0.1) |  |
| Crohn’s disease, n (%) | 61 (0.2) | 122 (0.1) | 0.018 | 50 (0.1) | 50 (0.1) |  |
| Burns and corrosions of external body surface, n (%) | 96 (0.3) | 270 (0.2) | 0.008 | 88 (0.2) | 81 (0.2) |  |
| NIHSS, n (%) | 2,139 (5.7) | 5,259 (4.3) | 0.066 | 1,962 (5.4) | 1,834 (5.0) |  |
| Echocardiography Procedures, n (%) | 2,065 (5.5) | 5,403 (4.4) | 0.052 | 1,915 (5.2) | 1,815 (5.0) |  |
| Cardiac Catheterization Procedures, n (%) | 377 (1.0) | 994 (0.8) | 0.021 | 346 (0.9) | 355 (1.0) |  |
| Electrocardiogram, routine ECG with at least 12 leads, n (%) | 6,836 (18.3) | 18,963 (15.4) | 0.076 | 6,377 (17.5) | 6,005 (16.5) |  |
| Antilipemic agents, n (%) | 7,480 (20.0) | 21,942 (17.9) | 0.055 | 7,164 (19.6) | 6,673 (18.3) |  |
| Beta blockers/related, n (%) | 7,229 (19.3) | 19,214 (15.6) | 0.097 | 6,857 (18.8) | 6,483 (17.8) |  |
| Antiarrhythmics, n (%) | 4,926 (13.2) | 13,670 (11.1) | 0.063 | 4,655 (12.8) | 4,345 (11.9) |  |
| Diuretics, n (%) | 4,237 (11.3) | 11,266 (9.2) | 0.071 | 3,984 (10.9) | 3,853 (10.6) |  |
| Calcium channel blockers, n (%) | 4,521 (12.1) | 13,387 (10.9) | 0.037 | 4,316 (11.1) | 4,068 (11.1) |  |
| Ace inhibitors, n (%) | 2,964 (7.9) | 9,577 (7.8) | 0.005 | 2,844 (7.8) | 2,715 (7.4) |  |
| Angiotensin II inhibitor, n (%) | 2,360 (6.3) | 7,810 (6.4) | 0.002 | 2,272 (6.2) | 2,091 (5.7) |  |
| Antianginals, n (%) | 1,669 (4.5) | 4,764 (3.9) | 0.029 | 1,590 (4.4) | 1,459 (4.0) |  |
| Anticoagulants, n (%) | 6,420 (17.2) | 13,536 (11.0) | 0.177 | 5,932 (16.3) | 5,664 (15.5) |  |
| Platelet aggregation inhibitors, n (%) | 6,067 (16.2) | 13,536 (11.0) | 0.036 | 5,814 (15.9) | 5,344 (14.6) |  |
| Alteplase, n (%) | 433 (1.2) | 1,189 (1.0) | 0.019 | 413 (1.1) | 386 (1.1) |  |
| Tenecteplase, n (%) | 99 (0.3) | 362 (0.3) | 0.006 | 97 (0.3) | 104 (0.3) |  |

ASD, absolute standardized mean difference; and HR, hazard ratio, NIHSS, National Institutes of Health Stroke Scale
